# Opioid prescribing and social deprivation: A retrospective analysis of prescribing for CNCP in Liverpool CCG

**DOI:** 10.1371/journal.pone.0280958

**Published:** 2023-03-08

**Authors:** Emma K. Begley, Helen M. Poole, Harry R. Sumnall, Bernhard F. Frank, Catharine Montgomery

**Affiliations:** 1 School of Psychology, Liverpool John Moores University, Liverpool, Merseyside, United Kingdom; 2 Public Health Institute, Liverpool John Moores University, Liverpool, Merseyside, United Kingdom; 3 Walton Centre NHS Foundation Trust, Liverpool, Merseyside, United Kingdom; 4 Pain Research Institute, University of Liverpool, Liverpool, Merseyside, United Kingdom; Friedrich-Alexander-Universität Erlangen-Nürnberg: Friedrich-Alexander-Universitat Erlangen-Nurnberg, GERMANY

## Abstract

**Background:**

Treating Chronic Non-Cancer Pain (CNCP) with long-term, high dose and more potent opioids puts patients at increased risk of harm, whilst providing limited pain relief. Socially deprived areas mapped from Index of Multiple Deprivation (IMD) scores show higher rates of high dose, strong opioid prescribing compared to more affluent areas.

**Objective:**

To explore if opioid prescribing is higher in more deprived areas of Liverpool (UK) and assess the incidence of high dose prescribing to improve clinical pathways for opioid weaning.

**Design and setting:**

This retrospective observational study used primary care practice and patient level opioid prescribing data for N = 30,474 CNCP patients across Liverpool Clinical Commissioning Group (LCCG) between August 2016 and August 2018.

**Method:**

A Defined Daily Dose (DDD) was calculated for each patient prescribed opioids. DDD was converted into a Morphine Equivalent Dose (MED) and patients stratified according to high (≥120mg) MED cut off. The association between prescribing and deprivation was analysed by linking GP practice codes and IMD scores across LCCG.

**Results:**

3.5% of patients were prescribed an average dose above 120mg MED/day. Patients prescribed long-term, high dose, strong opioids were more likely to be female, aged 60+, prescribed three opioids and reside in the North of Liverpool where there is a higher density of areas in the IMD most deprived deciles.

**Conclusion:**

A small but significant proportion of CNCP patients across Liverpool are currently prescribed opioids above the recommended dose threshold of 120mg MED. Identification of fentanyl as a contributor to high dose prescribing resulted in changes to prescribing practice, and reports from NHS pain clinics that fewer patients require tapering from fentanyl. In conclusion, higher rates of high dose opioid prescribing continue to be evident in more socially deprived areas further increasing health inequalities.

## Introduction

Chronic pain is a leading cause of disability worldwide and represents an emerging healthcare challenge and public health priority for many countries [1, 2]. In the UK, Chronic Non-Cancer Pain (CNCP) is currently estimated to affect between 30–50% of UK adults, with 10–14% reporting severe life-limiting pain [2]. Individuals living with CNCP are five times more likely than those without pain to access primary care health services, making it the most common health complaint [3, 4]. Effective treatments are limited [5] and symptoms of pain are commonly managed by prescription opioids.

Over the past 20 years a significant increase in opioid prescribing has been observed across Europe, North American and Australia [6–9]. In England for example, opioid prescribing increased from 228 million items in 1992 to 1.6 billion in 2009 and is currently estimated to cost the NHS over £300 million annually [10]). Although there was a slight decline between 2016–2017 attributed to a reduction in morphine, opioid prescribing has been on an upward trajectory [6, 11]. For example, prior to the rescheduling of Tramadol in 2014, it contributed to a surge in prescribing between 1995–2010 (0% in 1995 to 2.8% in 2010), with evidence of morphine, oxycodone, buprenorphine, and fentanyl also increasing 5-fold [12]. Similarly, Foy et al., (2016) found that weak opioids (such as codeine or tramadol) doubled over a 7-year period (2005–2012), compared to strong opioids (such as morphine or oxycodone) which increased by 6-fold [12]. Furthermore, during 2018 5.6 million adults living in England received at least one opioid, 540,000 of whom received continuous prescriptions between 2015–2018 [11].

The trajectory of increased opioid prescribing has prompted concern among healthcare professionals due to the lack of convincing evidence of their effectiveness long-term and exposure to heightened risk of harm [13–15]. Medium and long-term opioid use increases the risk of adverse effects such as constipation, nausea and dizziness [13], and likelihood of stepping up to stronger opioids or higher doses [12, 16]. To provide comparisons between different opioids, doses are usually converted into a summative Morphine Equivalent Dose (MED) [17]. For example, a large UK study reported an increased risk of adverse events such as falls, cognitive dysfunction, dependency, overdose, or death when doses above 120mg MED were taken long-term (>3 months) [15]. One US study of over 9,000 patients found that risk of overdose was 8.9 times more likely among patients taking daily doses above 100mg MED [18]. Furthermore, stronger opioids and higher doses have also been correlated with increased risk of opioid hyperalgesia [44], psychosocial problems (e.g. poor quality of life, loss of employment) [19] poor physical and mental health [20] and dependency [14]. UK clinical guidance suggests that there is no benefit of long-term high dose opioid prescribing and that patients prescribed doses exceeding 120mg MED a day should consider reducing or discontinuing treatment [21]. Recently, the latest NICE UK guidance recommended that opioids should no longer be prescribed to manage chronic primary pain, with a focus instead on using methods of self-management [22].

Previous research demonstrates that the majority of patients receiving long-term high dose opioid treatment are more likely to be: female; aged over 60 years; smokers; obese; depressed and living in areas of higher IMD quintile scores [8, 12, 15, 23–25]. This is thought to be driven partly by the higher prevalence of chronic pain in individuals with lower socioeconomic status (SES) [26, 27]. The link between IMD and prescribing has been noted in a number of studies [9, 23, 25, 28], where the divide between the North and South of England is reportedly one of the highest in Europe [29]. Mordecai and colleagues (2018) analysed prescriptions of eight opioids across 209 CCGs in England between 2010–2014 and found a significant positive relationship between MED and IMD score; this relationship was stronger when accounting for geography, suggesting greater MED in the north of England [9]. Similar disparities have also been reported by Chen et al., (2019) and Foy et al. (2016), who both found higher prescribing significant in areas of lower SES in Northern England [12, 25]. Moreover in Scotland, Torrance et al. (2018) found that strong opioid prescribing was 3.5 times more likely to occur in areas of higher deprivation [23]).

Risks of high dose opioid prescribing, and social deprivation add to the burden of chronic pain. The aim of the present study is to develop a profile of patients across LCCG prescribed high dose opioids, to assist in developing improved clinical pathways for opioid weaning. To achieve this, a scoping audit will be conducted to assess the incidence of high dose opioid prescribing in Liverpool, a northern city ranked the third most deprived local authority (LA) (out of 317) in England’s 2019 IMD scores.

## Method

### Setting and study sample

Ethical approval for this study was granted by LJMU Research Ethics Committee. A data sharing agreement for the extraction of patient level data was drawn up between LJMU and Liverpool CCG, in accordance with the Data Protection Act 2018. The inclusion criteria for patients were: age over 18 years; CNCP diagnosis; in receipt of any opioid prescription between August 2016 and August 2018. Patients with a history of substance dependence (current read code for dependence or free text entry indicating dependence in patient’s record), and those prescribed opioids to manage cancer pain (current read code for cancer diagnosis; prescribed opioid predominantly used in management of cancer pain not CNCP) were excluded. While we were interested in opioid prescribing for CNCP, the extracted data demonstrated that there was a great deal of heterogeneity in the coding of linked problems (providing reason why an opioid prescription was issued) with over 60,000 distinct reported problems. Categories of CNCP were created by grouping together similar conditions and conferring with a consultant anaesthetist (BF) to develop typologies. The most common linked problem for which opioids were prescribed was for musculoskeletal pain (n = 16,137) specifically back pain (n = 10,974) and arthritis (n = 7,154). For a full list of the 78 categories and frequency of linked prescriptions see S1 File. Upon further investigation of the linked problems, it was evident that there were anomalies in coding of linked problems, with some codes reflecting that a patient may have initially requested an appointment for an alternative reason. See S2 File for full list of linked problems.

### Study parameters, data extraction & filtering

**Fig 1:**
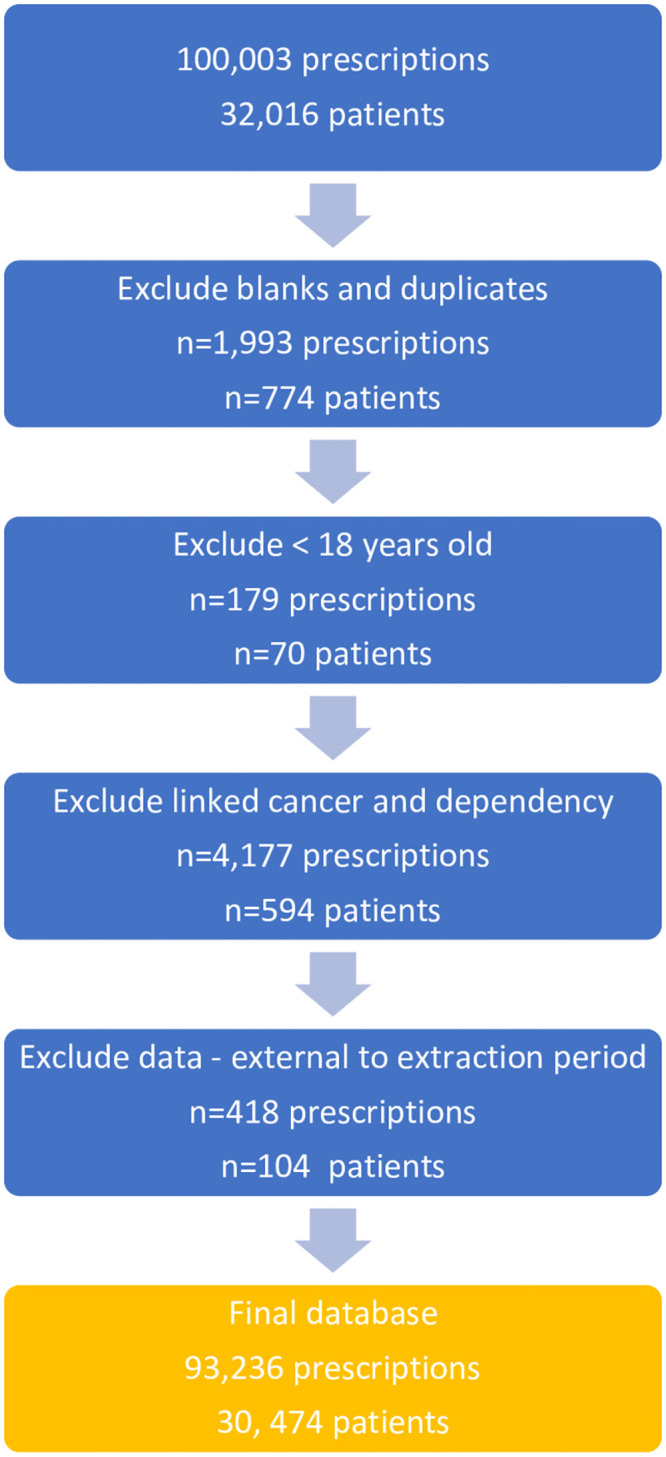
Data filtering and pre-processing pipeline.

The following data were extracted from patient Egton Medical Information System (EMIS) records: anonymised ID, age, ethnicity, gender, GP practice code, GP partial postcode, name of opioid, dose and quantity prescribed, date prescription was added to patient record, most recent issue date, course status (past or current) and any reported problems linked to the opioid prescription. Liverpool CCG (LCCG) acted as the gatekeeper and obtained verbal consent from GP practices to share patient information. Sixty-two of the 88 GP (70.5%) practices located across LCCG agreed to share patient data. An extract report was uploaded onto EMIS web, the data was extracted and then saved onto a secure network in an Excel spreadsheet. The data was pre-processed using Microsoft Excel, after which 93,236 prescriptions written for 30,474 patients remained (see Fig 1).

All prescriptions were cross-referenced with the British National Formulary (BNF) and re-coded according to their active opioid ingredient. This resulted in 12 groups including: oxycodone, tramadol, matazinol, methadone, morphine, tapentadol, pethidine, fentanyl, codeine, buprenorphine, dihydrocodeine and hydromorphine. Opioids commonly indicated for cancer or drug dependence (including dextropropoxphene, diamorphine, alfentanil, coproxomol, galenphol, oxylan and pavacol) were excluded. Dosage instructions were recoded to facilitate calculation of MED; if missing, maximal possible daily dose provided by the BNF was used.

### Calculation of Morphine Equivalent Doses (MED)

A Defined Daily Dose (DDD) for each prescription was calculated using the drug name and administration instructions; MED was calculated using DDD. Calculations for MEDs depended on the type of opioid prescribed and were computed using the equivalence parameters in Table 1, overseen by a Consultant Anaesthetist with extensive experience in opioid prescribing for CNCP (BF). The calculations needed to account for multiple daily opioid prescriptions that patients may take, whether or not they use the prescriptions concurrently. As a result, once MEDs were calculated for every prescription a new variable was created to calculate patients’ combined daily MED (MED sum) which reflects the total MED if they were to use all of their prescribed opioids. The purpose of this variable was to establish one potential total of MED for each patient, specifically for those with more than one prescription that may contribute to their daily morphine intake. However, it is clear that not all prescribed medication will be taken simultaneously, with patients choosing from a range of their prescribed medication according to the current severity of their pain. The new MED sum parameter was used to create an average MED variable, by dividing the MED sum by the total number of prescriptions for that patient, thus accounting for the multiple prescriptions that patients may receive. In summary, MED sum = the total potential MED for a patient based on all currently prescribed opioids and MED average = MED sum/number of currently prescribed opioids.

**Table 1:** Equivalence tables used in the calculation of DDD and MED.

	Morphine mg/24h					
	10	30	60	120	180	240
Oxycodone mg/24h	-	20*	40	80	120	160
Hydromorphone mg/24h	-	4	8	16	24	32
Methadone mg/24h	-	10	20	40	60	80
Fentanyl ug/h	-	-	12	25	-	50
Buprenorphine ug/h	-	10	20	40	52.2	70
Codeine mg/24h	100	240	-	-	-	-
Dihydrocodeine mg/24h	100	240	-	-	-	-
Tramadol mg/24h	67	200	400	-	-	-
Tapentadol mg/24h	25–50	75–150	150–300	300–600**	-	-

* Conversion used in USA, Canada & Australia

** The maximum recommended daily dose of Tapentadol prolonged release is 500mg.

For example, a patient prescribed co-codamol (30/500; 4 x 2 tablets per day), buprenorphine (10ug/h; 1 patch per week) and morphine sulphate (10mg/5ml; 2.5ml x 6 per day) would have a DDD of 240mg Codeine (30mg morphine) from the co-codamol, 10ug/hour (30mg morphine) from the buprenorphine and 30mg morphine from the morphine sulphate giving a total MED of 90mg (MED sum = 90). A patient may not take all of these medicines concurrently so prescriptions were averaged to give a daily MED average of 90/3 = 30mg MED.

### Statistical analysis

Data were analysed in IBM Statistical Package for the Social Sciences (SPSS) v26. Descriptive analysis identified the total number of patients, prescriptions issued and patient demographic data (sex, age and ethnicity). The proportion of patients prescribed an opioid, and multiple opioids at each GP practice were calculated by linking GP practice codes to available data for number of registered patients [30–32]. The data extracted from these sources was also used to develop two new variables distinguishing the neighbourhood and locality of each GP practice. Using these new variables and data for currently active prescriptions, the rate of prescribing was identified and reported in proportion to total registered patients at each practice. ANOVA was used to investigate differences in the percentages of patients prescribed opioids between localities (North, South and Central Liverpool).

Data were stratified into any current prescription in combination or standalone that exceeded 120mg MED/day, and separately where MED *average* doses exceeded 120mg/MED. Descriptive analysis provided an overview of types of opioids prescribed, number of patients and patient demographics linked to prescriptions exceeding 120mg/MED day in each GP practice. Percentages of the total number of prescriptions associated with doses exceeding 120mg/MED (N = 2,999) and those with average daily doses exceeding 120mg/MED (N = 601) were also calculated. We used these variables to investigate differences in prescribing across areas of LCCG.

## Results

A total of 93,236 opioid prescriptions were issued to 30,474 patients in primary care between 2016–2018. Most patients (40%) received only one opioid prescription during this time, however the number of prescriptions ranged from one to 82. Females represented 61% of this patient population (n = 18,580) and were slightly older than males (61 years ± 16.10 and 60 ± 14.77 years respectively). Most of the patients were identified as being white (78.60%), see Table 2 below.

**Table 2:** Patient demographics.

**Total N**^**o**^ **patients**	**30474**	
**Sex**	**18580 Female (61%)**	
**Mean Age (years)**	**61±16.10 (F)**	**60±14.77 (M)**
**Ethnicity**	**N (%)**	
White	23,953 (78.60%)	
British	23,125 (75.88%)	
Irish	246 (0.81%)	
Gypsy or Irish Traveller	10 (0.03%)	
Any other White Background	572 (1.87%)	
Mixed/Multiple ethnic groups	245 (0.81%)	
White and Black Caribbean	48 (0.16%)	
White and Black African	59 (0.20%)	
White and Asian	14 (0.05%)	
Any other Mixed/ Multiple ethnic background	124 (0.41%)	
Asian/ Asian British	487 (1.60%)	
Indian	61 (0.20%)	
Pakistani	59 (0.19%)	
Bangladeshi	30 (0.10%)	
Chinese	127 (0.42%)	
Any other Asian background	210 (0.69%)	
Black/ African/ Caribbean/ Black British	446 (1.46%)	
African	240 (0.78%)	
Caribbean	51 (0.17%)	
Any other black/African/Caribbean background	155 (0.51%)	
Other ethnic group	386 (1.27%)	
Arab	90 (0.30%)	
Any other ethnic group	296 (0.97%)	
Not disclosed	1,183 (3.88%)	
Not reported	3,774 (12.38%)	

The number of prescriptions issued by GP practices ranged from 207 to 4,510, but the proportion of patients within a practice prescribed an opioid varied greatly. Table 3 displays number and proportions of prescriptions issued at three of the highest and lowest prescribing practices. A comparison of GP practices 1–6 from Table 3 reveals that despite GPC01, 02, 04 and 05 having a similar number of registered patients, GCP04 and 05 prescribed opioids to fewer patients; n = 127 and n = 174 compared with the highest prescribing surgeries, n = 907 and n = 443 respectively. The three highest prescribing practices were all in IMD decile 1 (most deprived areas).

**Table 3:** Proportion of patients prescribed opioids at lowest vs. highest prescribing surgeries.

GP anonymised code	Locality	GP Practice postcode IMD Decile	Number of registered patients	Number of patients prescribed an opioid	Total number of opioid prescriptions issued	Proportion of total registered patients prescribed an opioid	Proportion of opioid prescriptions per patients registered
**GPC01**	North	1	6,680	907	3,169	0.14	0.47
**GPC02**	North	1	3,271	443	1,453	0.14	0.44
**GPC03**	North	1	5,112	487	2,255	0.10	0.44
**GPC04**	South	8	3,228	127	292	0.04	0.09
**GPC05**	Central	6	6,598	174	519	0.03	0.08
**GPC06**	Central	5	44,226	571	2,032	0.01	0.05

The following analyses describe current prescriptions which resulted in daily MEDs above 120mg during 2016–2018. In total, 1,069 patients (3.5% of the total sample) were in receipt of daily prescriptions >120mg MED, with 61/62 GP (98.4%) practices containing at least one patient >120mg MED, with doses above 120mg MED ranging between 124mg and 640mg MED. The majority of this subset were female (n = 710; 66%) and on average were older than males (58 years ±14.50 and 56 years ±12.62 respectively). The modal number of prescriptions was three per patient (range 1–14).

Fentanyl, oxycodone, buprenorphine, and morphine were the only single drugs issued in doses above the advised daily maximum dose (120mg MED). These drugs were also commonly prescribed simultaneously with other opioids, yielding an even higher daily dose. Morphine was most commonly prescribed in conjunction with other drugs that contributed to patients exceeding 120mg MED (see Table 4).

**Table 4:** Single opioid prescriptions compared with combination prescriptions >120mg MED.

Drug	No. of single prescriptions >120mg MED	No. of combination prescriptions equalling >120mg MED
Fentanyl	243	290
Oxycodone	148	525
Buprenorphine	121	282
Morphine	52	760

**Fig 2:**
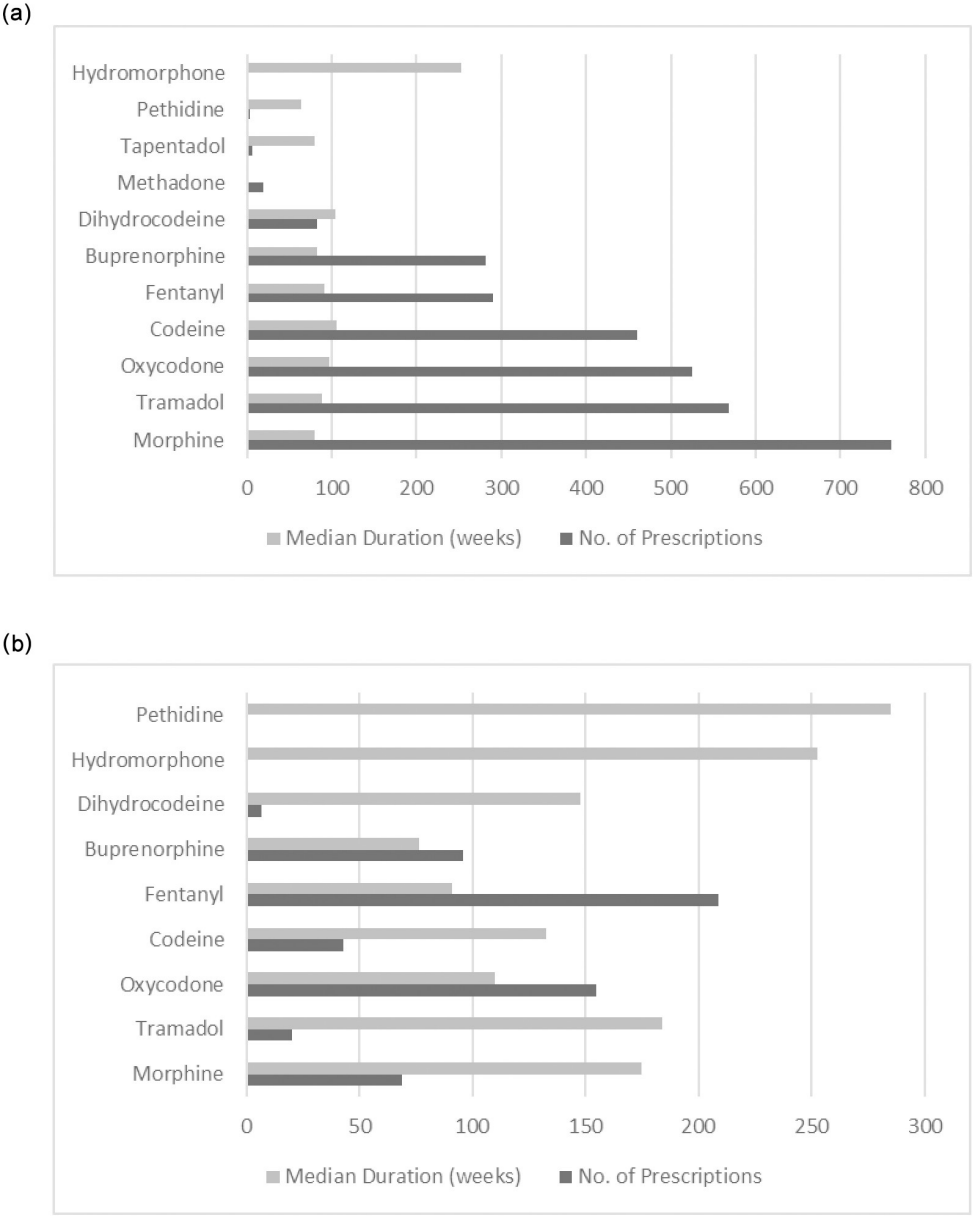
Average (Median) length of prescribing, and number of prescriptions for opioids commonly contributing to: a) Total MED doses >120mg/day. B) Average MED doses >120mg/day.

Prescriptions exceeding 120mg MED, either as single prescriptions or as a combination of prescriptions are shown in Fig 2a and 2b (see S3 File for median and range). These figures depict the number of prescriptions issued and the median duration an opioid was prescribed for daily doses above 120mg MED (Fig 2a), and average daily doses above 120mg MED (Fig 2b). Despite being the weakest opioid included in this analysis, codeine frequently contributed to patients’ overall daily dose.

Patients often receive more than one opioid and may not simultaneously take them, which is why *average* daily MEDs were calculated. We found 340 patients and 601 prescriptions, from 53 practices that prescribed average daily MED > 120mg MED (range 124mg-1120mg). Females continue to represent the majority of these patients 64% (n = 216) and were slightly older than males (61 years ±13.94 and 56 years ±12.23 respectively). Those patients in receipt of an *average* daily dose above 120mg MED were most commonly prescribed fentanyl (n = 171 patients, 35% (n = 209) prescriptions), followed by oxycodone (n = 83 patients, 26% (n = 155) prescriptions), buprenorphine (n = 74 patients, 16% (n = 96) prescriptions) and morphine (n = 48 patients, 11% (n = 69) prescriptions).

### Geographical differences in prescribing across Liverpool

There were 413,730 patients registered at the GP practices who were included in this analysis. Of these, 7.39% were prescribed an opioid and of those a fifth (21%) were issued more than one opioid. Table 5 displays the percentage of patients currently prescribed an opioid across the different localities in Liverpool. The highest rate of opioid prescribing as a percentage of practice population was found in north Liverpool, which has the highest proportion of IMD lower super output areas (LSOAs) in the highest deprivation decile compared to Central and South Liverpool. Levene’s test showed that the variance in % of patients in each locality prescribed an opioid were equal F(2,59) = 1.06, p = .35, and a Kolmogorov-Smirnoff test revealed that the data was normally distributed D(62) = .11, p = .08. ANOVA demonstrated a statistically significant difference in the percentages of patients prescribed an opioid between North, South and Central practices (F (2, 59) = 4.88, p = .01). Pairwise comparisons using Tukey HSD revealed that practices in north and south Liverpool differed significantly from each other (p = .02), while neither differed from central Liverpool.

**Table 5:** Percentage of patients currently prescribed an opioid across localities during 2016–2018.

	Average (mean) % of patients in a practice on an opioid (from practice population) ^1^	% no. of patients on >1 opioid (from practice population)	Of the patients currently prescribed an opioid what % are prescribed >1
**Citywide**	8%	1.5%	21%
**North**	9%	1.8%	20%
**Central**	7%	1.2%	23%
**South**	7%	1.4%	21%

^1^ Percentages are calculated using the total number of patients prescribed any opioid divided by the total number of registered patients in that GP locality. Percentage of patients on more than one opioid is calculated in the same manner. Patients prescribed more than one opioid as a percentage of those receiving any opioid is calculated as such: (patients prescribed >1 opioid/patients prescribed 1 opioid)*100.

**Fig 3:**
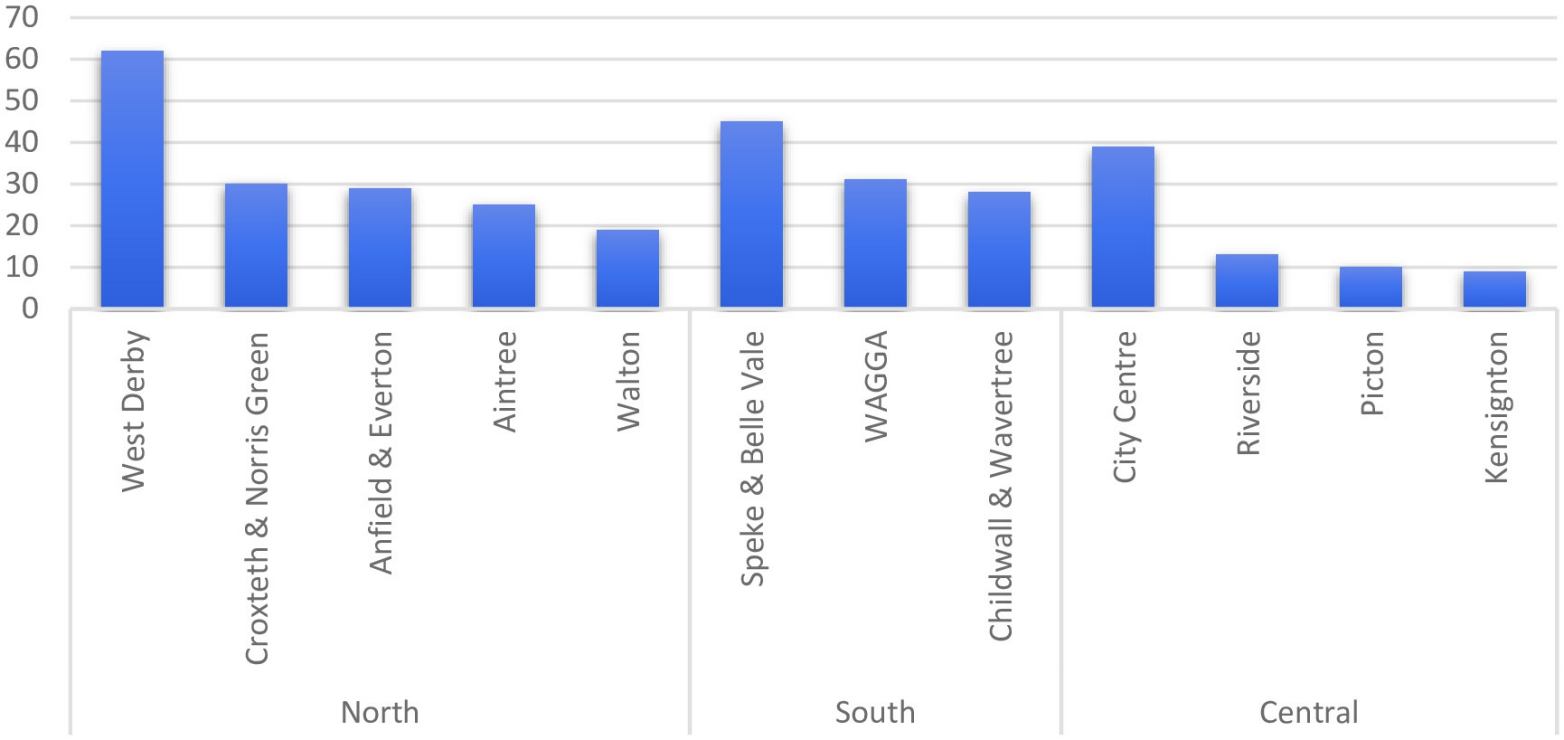
Locality and number of patients prescribed a daily average above 120mg MED.

Fig 3 highlights areas across the Liverpool CCG region where patients were in receipt of opioids prescribed above a daily average of 120mg MED. GP practices in the North of Liverpool prescribed high doses to the highest number of patients. Neighbourhoods across South Liverpool had relatively similar prescribing practices to those in the North.

## Discussion

We analysed opioid prescription data from 62 (out of 83) GP practices across LCGG between August 2016 –August 2018. During this period, 93,236 opioid prescriptions were issued to 30,474 patients. Most patients were female, aged around 60 years, identified as white British and were commonly prescribed one opioid, with doses below 120mg MED/day. This is consistent with other national cross-sectional studies that highlight increased prevalence of weaker opioids [23], predominance of low daily doses [8], patients being mostly female [12, 20], and generally older adults [25]. This data implies that most GPs were prescribing within the recommended limits. There was, however, a small but significant proportion of patients (n = 1,069; 3.5%) being prescribed opioids above 120mg MED over the long-term, perhaps indicating that these patients require support and intervention to help reduce their opioid use and optimise their pain treatment.

Systematic reviews [14, 19], empirical research studies [15] and national clinical guidance [33] have all reiterated the lack of efficacy and increased risk of harm of long-term opioid use, particularly when daily doses are above 120mg MED [34, 35]. National trends in opioid prescribing in primary care [36–38], suggest that an increase in prescribing and corresponding risk of harm to CNCP patients warrants attention [8, 9, 23, 25]. Our findings were in keeping with other recent analyses. In Scotland during 2018, codeine was the most frequently prescribed opioid, followed by tramadol and then morphine [23]. For the past six years morphine has remained the most frequently prescribed high strength opioid in the UK [8, 9, 23]). Prescribing trends across the UK have also consistently shown that although stronger opioids such as fentanyl, oxycodone or buprenorphine are less frequently prescribed than weaker opioids such as codeine or tramadol, trends of strong opioid prescribing are increasing year on year [8, 12, 23, 24]. Our data showed that strong opioids (fentanyl, oxycodone, buprenorphine, and morphine) were less frequently prescribed but contributed to patients exceeding 120mg MED/day.

Other prevalence studies have focused on these stronger opioids [8, 9, 24], but do not identify specific combinations contributing to high daily doses. For example, Dunn (2010) and Bedson et al. (2019) found that doses exceeding 100mg MED/day were attributed to at least three opioids and significantly increased patients’ risk of fracture, falls, overdose, and death [15, 18], but there is a gap in the literature identifying which combinations of opioids are most likely to contribute to doses above 120mg MED/day. Similarly, patients in this study receiving prescriptions above 120mg MED/day were most likely to receive three opioids (modal response). However, we also identified that whilst morphine prescribed on its own was least likely to exceed 120mg MED; in combination with other opioids, it was almost 14 times more likely to exceed this dose. Furthermore, the higher the daily dose of morphine the longer the duration of the prescription (findings similar to Foy et al., 2016).

The present study confirms the relationship between opioid prescribing and deprivation, highlighting that GPs in more socially deprived neighbourhoods in north Liverpool, were more likely to prescribe high doses of opioids (9%) compared to South (7%) and Central Liverpool (7%). Additionally, patients in North Liverpool were more likely to receive more than one opioid, compared to patients from practices located in South and Central Liverpool (1.8%, 1.2% and 1.4%, respectively). Some of the higher prescribing practices in the North and South of Liverpool display the highest levels of deprivation in the city [39]. It is not clear whether the differences across these areas are due to prescribing practices or different patient health needs, nor are these factors mutually exclusive. Todd (2018) argues that a number of compositional (e.g. patient demographic, SES, health behaviours), contextual (e.g. stigma, access to services, employment) and co-morbidity (e.g. anxiety and depression) factors contribute to the differences in pain and prescribing [28]. Mordecai et al (2018) suggest that it is perhaps due to the higher prevalence of chronic pain reported in people living in areas of higher deprivation [9]. Even after controlling for deprivation, Jani *et al* (2020) found disparities in prescribing between the North and the South of England indicating greater health care needs in the North [16]. Jani *et al* (2020) also demonstrated that a minority of individual prescribers (3.5%) contributed to the small proportion of high prescribing practices (25.6%) and the likelihood of patients continuing a long-term opioid prescription [16]. It is likely that the increase in opioid prescribing is driven by a combination of all these factors, and the current study indicates the need to ensure that clinical guidance is implemented in practices based in areas of higher deprivation, perhaps via targeted work on adherence in these high prescribing practices.

At a local level, this study identified that there is a small cohort of patients who should be prioritised for treatment review. The characteristics around the prescribing practices of these patients could be used to identify other potential patients at risk of inappropriate prescribing and facilitate intervening before it occurs or escalates. It is equally important however, that risk of harm doesn’t deter prescribers from issuing opioids altogether, as at lower doses they are arguably effective for CNCP among some patients groups (e.g. those who experience fewer side effects) [40]. To strike this balance of minimising risk and maximising benefit, a pro-active approach to prescribing has been recommended, this requires prescribers to closely monitor, review and risk assess patients throughout their opioid treatment [41]. There is evidence of the effectiveness of interventions reviewing and supporting GPs, and delivering regular bespoke feedback regarding opioid prescribing, with the development of the Campaign to Reduce Opioid Prescribing [42] demonstrating that regular comparative feedback to GP practices over a year resulted in reductions in prescribing of strong opioids, total opioid prescriptions and high-risk prescribing. The same group found that the feedback was positively received by GPs and the feedback allowed practices to develop strategies consistent with their own priorities [43]. Taken together, these studies give recommendations for a clinician based intervention which could complement patient-based weaning support. There is evidence that targeted weaning support programmes are effective in both reducing opioid use and improving other indicators of quality of life [44] and utilising these programmes in high prescribing practices should be a priority. In addition, the results from the present study have been used to revise the guidance on the Pan Mersey Formulary, moving Fentanyl from amber to red list, indicating that it can only be prescribed by specialist pain services. Local NHS pain clinics have noted (personal communication with BF) that fewer patients present to pain clinics on high dose fentanyl as a result of this.

The majority of UK studies describe prescribing trends at national and regional levels, and so a key strength of this study rests in the presentation of individual practice and patient level data and its relation to areas of social deprivation. The study is also representative of patients across LCCG, with 62 out of 83 practices agreeing to share their data and has identified a number of different prescribing trends and common practices which complements and extends the published literature. There are however a number of limitations. Firstly, while all patients were coded in EMIS as having CNCP, patients may present to clinical appointments with numerous problems; therefore, linked problems in EMIS may not always reflect the CNCP diagnosis (e.g. *driving licence application*). Some patient records were incomplete such as ethnicity, linked problem or advised dosing instructions. The latter was compensated by presuming the highest dosing instruction advised from the BNF and may account for an over or under estimation in some of the calculated MEDs. Whilst carrying out the analysis for this study it became clear that prescribing data must be interpreted cautiously. For example, patients may be issued brief prescriptions or exceptions to their usual prescription (reasons for which are unknown). As a result, on record this would appear to increase a patient’s daily dose even though they may not take all prescriptions simultaneously. While these could be excluded as outliers, the nature of treating chronic pain means that patients do frequently receive multiple prescriptions, as such, an average MED was calculated and patients still exceeding 120mg MED/day were identified. The data did not allow us to assess what proportion of patients started on 120mg MED as the EMIS system only recorded current dosing instructions for each medication; future research should seek to use time series analyses to investigate prescribing in individual patients over time. Limitations of the study design meant that the time frame of data extraction is shorter than some of the published literature which prevented a time trend analysis. In addition, the project timeframe also meant that we could not calculate an exact duration for each prescription as some start/end dates were often outside of our data collection window. The data also does not provide a reason for differences in practice prescribing patterns and what happens at patient-doctor level. More understanding of this would require in-depth qualitative research to investigate the experiences of doctor-patient prescribing practices. Lastly, it is difficult to know if prescriptions were dispensed and used by patients, without further evidence linking prescriptions to dispensaries and feedback from patients.

Due to the mounting evidence that opioid related harm is dose dependent [14, 18, 44], it is concerning that of the 3.5% of patients exceeding 120mg MED/day, and that 34% (n = 360) of them received *average* daily dose above this threshold. The key characteristics associated with high dose prescribing that were identified here could be used to identify patients for review. The British Pain Society (BPS) recommends that patients prescribed doses above 120mg MED/day should be referred to specialist pain clinics for additional support [45]. However, the capacity in specialist pain clinics is already limited and this calls for more accessible interventions within the community. Future research should consider stratifying patients at a community level who are receiving high dose opioids and in need of interventions designed to optimise their chronic pain treatment.

## Supporting information

S1 File Categories of reported CNCP and frequency of prescriptions. (DOCX)

S2 File Grouped reasons for health care visit and prescription. (DOCX)

S3 File Average length of opioid prescriptions. (DOCX)
