# Supplementary material for: Opioid prescribing and social deprivation: A retrospective analysis of prescribing for CNCP in Liverpool CCG
Source: PLoS One. 2023 Mar 8;18(3):e0280958. doi: 10.1371/journal.pone.0280958 (PMC9994720; doi:10.1371/journal.pone.0280958)
Supplement: S1 File — (DOCX) [file pone.0280958.s001.docx]

# Supplementary file S1:

# Categories of reported CNCP and frequency of prescriptions

| **CNCP** | **No. of prescriptions** |
| --- | --- |
| Musculoskeletal pain | 16,137 |
| Back Pain | 10974 |
| Arthritis | 7154 |
| Mental Health | 2169 |
| skin complaints | 1488 |
| Respiratory problems | 1248 |
| General aches and pains | 1122 |
| Abdominal pain | 1060 |
| Urinary system complaints | 1039 |
| Infection | 1014 |
| Medication Review | 1006 |
| Headache | 924 |
| Gastroenterology Problems | 899 |
| Medication requested | 885 |
| Accident or Fall | 863 |
| Surgery/treatment | 840 |
| ENT Complaint | 762 |
| Gynaecological or reproductive issues | 692 |
| Cough | 671 |
| Hypertension | 622 |
| Blood Deficiency | 507 |
| Bowel Dysfunction | 466 |
| Neuropathy | 414 |
| Diabetes | 346 |
| Osteoporosis | 328 |
| Prosthetic replacement | 310 |
| Tiredness or sleep | 299 |
| Swelling/inflammation | 246 |
| Blood Vessel Conditions | 244 |
| Consultation | 244 |
| Heart Condition | 229 |
| Testing | 205 |
| Lump on body | 189 |
| Spinal stenosis | 181 |
| Brain dysfunction | 180 |
| Sinusitis | 176 |
| Chronic Intractable pain | 171 |
| Endometriosis | 153 |
| Shingles | 146 |
| Weight issue | 145 |
| Vertigo | 144 |
| Not medically related | 138 |
| Repeat Prescription | 137 |
| Eye Complaint | 117 |
| Dental Complaints | 114 |
| Groin discomfort | 111 |
| Assault | 91 |
| Adverse reaction/allergic reaction | 77 |
| Cramping | 74 |
| Wound care | 73 |
| Chronic Regional Pain Syndrome | 72 |
| Other | 72 |
| Viral illness | 71 |
| Facial Pain | 69 |
| Chest Discomfort | 65 |
| Thyroid Issues | 63 |
| Hormone replacement | 61 |
| Fever | 58 |
| Multiple symptoms | 57 |
| Flu | 52 |
| Malaise | 52 |
| Pins and Needles | 51 |
| Therapeutic Prescription | 49 |
| Tumour | 47 |
| Referral | 44 |
| Male Genitourinary Tract | 42 |
| Whiplash injury | 36 |
| Memory | 31 |
| Smoking | 26 |
| Lymphadenopathy | 22 |
| Lupus | 21 |
| Numbness | 18 |
| Supportive care | 18 |
| Burning sensation | 15 |
| Seizure | 15 |
| Spina Bifida | 15 |
| Ingrowing toe nail | 13 |
| Suspected condition | 12 |
