# Supplementary material for: Opioid prescribing and social deprivation: A retrospective analysis of prescribing for CNCP in Liverpool CCG
Source: PLoS One. 2023 Mar 8;18(3):e0280958. doi: 10.1371/journal.pone.0280958 (PMC9994720; doi:10.1371/journal.pone.0280958)
Supplement: S2 File — (DOCX) [file pone.0280958.s002.docx]

# Supplementary file S2: Grouped reasons for health care visit and prescription

- Abdominal Pain

Abdominal lump, Abdominal mass, Abdominal pain, Abdominal swelling, Epigastric pain, Hypochondrial pain, Nonspecific abdominal pain, Pain in left iliac fossa, Recurrent acute abdominal pain, Right lower quadrant pain, Upper abdominal pain, Sphincter of Oddi, Spinal nerve root, AAA - Abdominal aortic aneurysm without mention of rupture, Abdomen feels bloated, Abdomen feels distended, Abdominal discomfort, Abdominal distension symptom, Abdominal pain, Abdominal pain type, Abdominal wall pain, Colicky abdominal pain, Dysmenorrhoea, Epigastric hernia, Epigastric pain, Examination of abdomen, Flatulence/wind, Flatulent dyspepsia, Hiatus hernia, Hiatus hernia NOS, Lower abdominal pain, Neoplasm of uncertain behaviour of retroperitoneum, abdomen pain hypochondrium, Paraumbilical hernia, Peritoneal adhesions, Primary dysmenorrhoea, Primary repair of incisional hernia, Primary repair of inguinal hernia, Right iliac fossa pain, Right inguinal hernia, Sliding hiatus hernia, Spasm of sphincter of Oddi, Spigelian hernia, Suprapubic pain, Umbilical hernia, Upper abdominal pain, Ventral hernia, Wind symptom

- Accident or fall

Accidental injury, Motorcycle rider injury in collision with car, pick-up truck or van, RTA - Road traffic accident, RTA - Road traffic and other transport accidents, Unspecified fall, Abrasion, lower leg, Accidental fall, Accidental falls, Accidental injury NOS, Closed crush injury, hip, Closed crush injury, shoulder area, Closed injury, suprascapular nerve, Crush injury, Crush injury of arm, Crush injury, elbow, Crush injury, finger(s), Crush injury, foot, Crush injury, lower limb, Crush injury, trunk, Fall – accidental, Fall - accidental tripped over paving stone (GMS), Fall on or from stairs or steps, Fall on same level from slipping, tripping or stumbling, Fall on same level from tripping, Falls, head injury, Had a collapse, Head injury, History of road traffic accident, Injury arm, Injury NOS, Injury of lower leg, Injury toe, Minor head injury, Motor vehicle accident, Motor vehicle traffic accidents (MVTA), MVTA-unspecified - pedestrian injured, Other falls, Other motor vehicle traffic accident with collision on road, Other road vehicle accidents, Post-concussion syndrome, Recurrent falls, RTA - motor vehicle, RTA - motor vehicle-4 days ago side impact-car (GMS), RTA injury examination, Unspecified injury of hand, Unspecified injury of wrist.

- Adverse medical reaction/allergic reaction

Accident poison/exposure to narcotic drug at home, Accidental drug overdose / other poisoning, Adverse drug reaction NOS, Adverse reaction to Butrans, Adverse reaction to Matrifen, Adverse reaction to Pregabalin, Adverse reaction to Tramadol Hydrochloride, Adverse reaction to vaccine or biological substance NOS, Allergic reaction, Anaphylactic shock, Dr stopped drugs - side effect, Drug declined by patient - side effects, Drug not taken - side-effects, Drug withdrawal syndrome, FH: Hay fever, drug allergy, hay fever, Has shown side effects from medication, Hay fever, Hay fever - other allergen, Hay fever – pollens, Hay fever - unspecified allergen, Irradiation hypothyroidism, Medication stopped - side effect.

- Alcohol issue

Problems related to lifestyle alcohol use, Alcohol abuse – nondependent, Alcohol dependence syndrome, Alcohol detoxification, Alcohol problem drinking, Alcohol-induced chronic pancreatitis, Alcoholic cirrhosis of liver, Alcoholic fibrosis and sclerosis of liver, Alcoholic hepatitis, Alcoholic liver damage unspecified, Alcoholism, Binge drinker, Chronic alcoholism, Excessive use of alcohol, Nondependent alcohol abuse unspecified, Referral to specialist alcohol treatment service.

- Arthritis

Inflammatory polyarthropathies, Other specified arthritis, Seropositive rheumatoid arthritis unspecified, Acute polyarticular juvenile rheumatoid arthritis, Ankle arthritis NOS, Ankle osteoarthritis NOS, Arthritis, ARTHRITIS, Arthritis - lumbosacral (GMS), Arthritis – spine, Arthritis associated with other disease, IP joint of toe, Arthritis of spine, Arthritis/arthrosis, Arthropathies and related disorders, Arthropathies NOS, Arthropathy – psoriatic, Arthropathy (GMS), Arthropathy NOS, Arthropathy NOS-hand, Arthropathy NOS, of multiple sites, Arthropathy NOS, of the ankle and foot, Arthropathy NOS, of the shoulder region, Arthropathy NOS, of unspecified site, Arthroscopic debridement of knee joint, Arthrosis of first carpometacarpal joint unspecified, Chronic arthritis, Elbow arthritis NOS, Elbow joint pain, Elbow osteoarthritis NOS, Erosive osteoarthrosis, Finger osteoarthritis NOS, Flare of rheumatoid arthritis, Foot arthritis NOS, Foot osteoarthritis NOS, Generalised arthritis, Generalised osteoarthritis – OA, Generalised osteoarthritis NOS, Generalised osteoarthritis of multiple sites, Generalised osteoarthritis of the hand, Generalised osteoarthritis-OA, Gout, GOUT, Gout NOS, Gouty arthritis, Gouty arthropathy, Gouty arthropathy, arthritis, osteoarthritis, rheumatoid arthritis, Hand arthritis NOS, Hip arthritis NOS, Hip osteoarthitis NOS, Hip osteoarthritis NOS, Inflammatory polyarthropathy, Inflammatory polyarthropathy NOS, Juvenile rheumatoid arthritis, Juvenile rheumatoid arthritis NOS, Knee arthritis NOS, Knee osteoarthritis NOS, Localised osteoarthritis, unspecified, NOS, Localised osteoarthritis, unspecified, of shoulder region, Localised osteoarthritis, unspecified, of the ankle and foot, Localised osteoarthritis, unspecified, of the hand, Localised osteoarthritis, unspecified, of the lower leg, Localised osteoarthritis, unspecified, pelvic region/thigh, Localised, primary osteoarthritis, Localised, primary osteoarthritis of elbow, Localised, primary osteoarthritis of the hand, Localised, primary osteoarthritis of the lower leg, Osteoarthritis, OSTEOARTHRITIS, Osteoarthritis - ankle/foot, Osteoarthritis - elbow joint, Osteoarthritis - hand joint, Osteoarthritis - hip joint, Osteoarthritis – knee, Osteoarthritis - knee joint, Osteoarthritis - knee joint, Osteoarthritis - knee joints, Osteoarthritis - knee joints, Osteoarthritis – NOS, Osteoarthritis - other joint, Osteoarthritis – spine, Osteoarthritis - wrist joint, Osteoarthritis -multiple, Osteoarthritis -multiple joint, Osteoarthritis -shoulder joint, Osteoarthritis (GMS), Osteoarthritis and allied disorders, Osteoarthritis cervical spine, Osteoarthritis NOS, Osteoarthritis NOS-hand, Osteoarthritis NOS, of 1st MTP joint, Osteoarthritis NOS, of acromioclavicular joint, Osteoarthritis NOS, of ankle, Osteoarthritis NOS, of ankle and foot, Osteoarthritis NOS, of elbow, Osteoarthritis NOS, of hip, Osteoarthritis NOS, of knee, Osteoarthritis NOS, of PIP joint of finger, Osteoarthritis NOS, of shoulder, Osteoarthritis NOS, of shoulder region, Osteoarthritis NOS, of subtalar joint, Osteoarthritis NOS, of the hand, Osteoarthritis NOS, of the lower leg, Osteoarthritis NOS, of unspecified site, Osteoarthritis NOS, of wrist, Osteoarthritis NOS, other specified site, Osteoarthritis NOS, pelvic region/thigh, Osteoarthritis of cervical spine, Osteoarthritis of knee, Osteoarthritis of lumbar spine, Osteoarthritis of spinal facet joint, Osteoarthritis of spine, Osteoarthritis of thoracic spine, Osteoarthritis spine, Osteoarthritis, back, Osteoarthritis+allied disord, OSTEOARTHROSIS, OSTEOARTHROSIS FINGERS, OSTEOARTHROSIS KNEE(S), STEOARTHROSIS SPINE, Other juvenile arthritis, Palindromic rheumatism, PATELLO FEMORAL ARTHRITIS, Patellofemoral osteoarthritis, Pauciarticular juvenile rheumatoid arthritis, Periarthritis NOS, Periarthritis of shoulder, Periorbital oedema,Polyarthritis, Polyarthritis NOS, Polyarthritis NOS (GMS), Polyarthropathy NEC, Primary generalized osteoarthrosis, Pseudogout, Psoriatic arthritis, Psoriatic arthropathy, Psychiatric monitoring, Pyogenic arthritis of the pelvic region and thigh, Rheum. arth. - knee joint, Rheum. arth. - multiple joint, Rheumat.dis.- joints affected, Rheumatic pain, Rheumatism NOS – multiple, Rheumatism unspecified, Rheumatism/fibrositis NOS, Rheumatoid arthrit monitoring, Rheumatoid arthritis, RHEUMATOID ARTHRITIS, Rheumatoid arthritis - multiple joint, Rheumatoid arthritis annual review, Rheumatoid arthritis monitoring invitation first letter, Rheumatoid Arthritis NOS, Rheumatol. disorder monitoring, Septic arthritis, Sero negative arthritis, Sero negative polyarthritis, Sero-Negative Polyarthritis, Seronegative rheumatoid arthritis, Seropositive rheumatoid arthritis, unspecified, Shoulder arthritis NOS, Suspected inflammatory arthritis, Thumb osteoarthritis NOS, Toe osteoarthritis NOS, Undifferentiated inflammatory arthritis, Unspecified polyarthropathy of multiple sites, Unspecified polyarthropathy or polyarthritis, Unspecified polyarthropathy or polyarthritis NOS, Wrist arthritis NOS, Wrist osteoarthritis NOS

- Asperger’s

Asperger's syndrome

- Assault

Domestic violence, Victim of crime and terrorism, Assault by fight, Assault, Assault - gun, larger gun, Assault by bodily force, Assault by bodily force, occurrence at home, Stabbing, Accident caused by gunshot wound NOS, Assault by cutting and stabbing instruments, Assault by unspecified means, At risk of domestic violence, Dog bite, Gunshot accident, History of domestic violence, Suspected assault - allegation made, Victim of domestic violence.

- Back pain

Central disc prolapse, Lateral spinal stenosis, Spondylolisthesis grade 1, Spondylolisthesis grade 2, Chronic back and neck problems, Facet joint of lumbar spine, Spinal nerve root, Spinal nerve root C5, Spinal nerve root L5, Spinal nerve root of lumbar spine, Spinal nerve root S1, Lower back injury, Other forms of scoliosis, Spondylopathies, Lumbar spine - no cord lesion, Abnormal Lumbar Spine, Acquired kyphosis, Acquired kyphosis NOS, Acquired spondylolisthesis, Acute back pain – lumbar, Acute back pain – thoracic, Acute back pain – unspecified, Acute back pain + sciatica, Acute back pain with sciatica, Anterior spinal and vertebral artery compression syndromes, Arachnoiditis, Back disorders NOS, back pain, Back pain, BACK PAIN, Back pain . has tender left, Back pain and sciatica left, Back pain – lower, Back pain - wedge fracture, Back pain 10 years at least, Back pain 12/12 at least no, Back pain 3 years MRI scan, Back pain chronic, Back pain CHRONIC, Back pain extensive, Back pain investigated at, Back pain no previous, Back pain off and on 5, Back pain scoliosis, ch back, Back pain since 93, Back pain since94, Back pain some, Back pain without radiat NOS, Back pain without radiat NOS-accident (GMS), Back pain without radiation NOS, Back pain, restricted, Back pain, restricted spinal, Back pain, spinal movement, Back pain unspecified, Back sprain, Back sprain NOS, Back stiffness, Backache, Backache (GMS), Backache and neck pain, ex, Backache low, Backache symptom, Backache treated with, Backache with radiation, Backache with radiation down, Backache, Chronic. Backache, discussed, Backache, unspecified, Backache, Backache. Isq, low back pain, lumbar pain, upper back ache, Chronic low back pain, Coccygodynia, Collapse of lumbar vertebra, Congenital kyphosis, Congenital lumbosacral spondylolysis, Congenital spondylolisthesis, Decompression of spine NOS, Degeneration of lumbar spine, Degenerative cervical spinal stenosis, Degenerative disc disease NOS, Degenerative lumbar spinal stenosis, Degenerative spondylolisthesis, Denervation of spinal facet joint of lumbar vertebra NEC, Disc prolapse with myelopathy, Facet joint syndrome, back problem, Idiopathic scoliosis, Intervertebral disc disorders, Intervertebral disc prol. NOS, Intervertebral disc prolapse NOS, Kyphoscoliosis and scoliosis, Kyphoscoliosis or scoliosis NOS, LBP - low back pain, Low back pain, Lumbago, Lumbago with sciatica, Lumbalgia, Lumbar ache – renal, Lumbar back sprain, Lumbar back sprain (GMS), Lumbar disc degeneration, Lumbar disc disorder with myelopathy, Lumbar disc displacement, Lumbar disc displacement without myelopathy, Lumbar disc lesion – displaced, Lumbar disc lesion - displaced (GMS), Lumbar disc prolapse with cauda equina compression, Lumbar disc prolapse with myelopathy, Lumbar disc prolapse with radiculopathy, Lumbar discitis, Lumbar DXA scan result osteopenic, Lumbar DXA scan result osteoporotic, Lumbar spinal stenosis, Lumbar spondylosis, Lumbar spondylosis (GMS), Lumbar sprain, Lumber disc bulge, Lumbosacral spond + myelopathy, Lumbosacral spond-no myelopath. (GMS), Lumbosacral spondylosis with radiculopathy, Lumbosacral spondylosis without myelopathy, Lumbosacral sprain, Lumbosacral strain, Mechanical low back pain, Mechanical low back pain (Synergy code: @16CA), Multiple joint pain. ch back, Myelopathy NOS, Nerve root and plexus compressions in spondylosis, O/E - spine abnormal NOS, Osteoarthritis back&shoulder, Osteoarthritis, ch backache, Other and unspecified back disorders, Other back injuries, Other lumbar disc disorders, Pain In Back, Paraplegia, PID - prolapsed lumbar disc, Postural scoliosis, Prolapsed intervertebral disc without myelopathy, Prolapsed lumbar intervertebral disc, Prolapsed lumbar intervertebral disc with sciatica, Pulled back muscle, Sacrococcygeal sprain, Scheuermann's disease, Sciatica, SCIATICA, Sciatica rt, Scoliosis – acquired, Scoliosis associated with other condition, Scoliosis of thoracic spine, Single-level cervical spondylosis without myelopathy, Slipped intervertebral disc, Spasm of back muscles, Spinal cord compression, Spinal cord compression NOS, Spinal disorder NOS, Spinal injuries, Spondylitis NOS, Spondylolisthesis (GMS), Spondylolysis, Spondyloses, Spondylosis + allied disorders, Spondylosis and allied disorders, Spondylosis NOS, Sprain of other parts of back, Thoracic back pain, Thoracic disc degeneration, Thoracic discitis, Thoracic spondylosis, Vertebral column syndromes, Wedge compression # lumbar spine, Wedge Compression # Lumbar Spine, Wedge compression # of dorsal spine, Wedge Compression # Of Dorsal Spine

- Blood deficiency

Alpha trait thalassaemia, Anaemia unspecified, Anticoagulant therapy, Antiphospholipid syndrome, Aortic aneurysm, B12 injections - at surgery, Blood dyscrasia NOS, Blood pressure monitoring, Borderline blood pressure, Chronic venous insuffic.NOS, Chronic venous insufficiency NOS, Combined B12 and folate deficiency anaemia, ESR raised, Ferritin level low, Folate-deficiency anaemia, Folic acid deficiency, raised blood lipids, Haemoglobin estimation, Haemoglobin low, Haemoptysis – symptom, Hypercholesterolaemia, Hypercholesterolaemia (GMS), Hyperkalaemia, Hyperlipidaemia, Hyperlipidaemia NOS, Hyperparathyroidism, Hyperprolactinaemia, Hypocalcaemia NEC, Hyponatraemia, Idiop thrombocytopenic purpura, Immunoglobulins, Impaired glucose regulation, Impaired glucose tolerance, Intramuscular injection of vitamin B12, Iron deficiency, Iron deficiency anaemia NOS, Iron deficiency anaemias, ITP - idiopathic thrombocytopenic purpura, Lymphocytosis, Lymphoedema, Macrocytic anaemia unspecified cause, Macrocytosis - no anaemia, Microcytic - hypochromic anaemia, Microcytic hypochromic anaemia, Mixed hyperlipidaemia, Monoclonal gammopathy of uncertain significance, Myelodysplastic and myeloproliferative disease, Neutropenia, Neutrophilia, Perthe's disease, Perthes' disease - osteochondritis of the femoral head, Plasma factor V level, Plasma testosterone level, Possible familial hypercholesterolaemia, Pure hypercholesterolaemia, Pure hypercholesterolaemia NOS, Secondary anaemia NOS, Serum cholesterol, Serum cholesterol raised, Serum cholesterol very high, Serum cortisol, Serum digoxin level, Serum ferritin high, Serum folate low, Serum iron level, Serum iron low, Serum testosterone, Serum triglycerides raised, Serum vitamin B12, Serum vitamin B12 low, Serum vitamin D, Serum vitamin D -Req, Serum zinc level low, Thrombophilia, Thrombophlebitis migrans, Thrombophlebitis NOS, Thrombophlebitis of a superficial leg vein NOS, Thrombosis of vein of leg, Vitamin B12 deficiency, Vitamin B12 deficiency anaemia, Vitamin D deficiency, Vitamin D insufficiency, Vomiting, Vomiting symptoms, White cell count.

- Blood vessal conditions

Cavernous haemangioma, Superficial femoral artery, Atherosclerosis, Behcet's syndrome, Behcets syndrome, Buerger's disease, Chilblains, Chronic peripheral venous hypertension, Deep vein thrombosis, Deep vein thrombosis leg, DVT - Deep vein thrombosis, DVT - not obstetric x 2 over past 2 yrs on, Embolism and thrombosis of the radial artery, False aneurysm, Familial, hypercholesterolaemia, Giant cell arteritis, Giant cell arteritis with polymyalgia rheumatica, Granulomatosis with polyangiitis, Deep Vein Thrombosis, Hereditary haemorrhagic telangiectasia, Ischaemia of legs, Ischaemic foot, Melaena, Nonpyogenic venous sinus thrombosis, Normocytic anaemia due to unspecified cause, phlebitis, pompholyx, Occlusion of posterior tibial artery, Oesophageal varices, Other peripheral vascular dis, Other specified peripheral vascular disease, Peripheral arterial disease, Peripheral ischaemia, Peripheral ischaemic vascular disease, Peripheral vascular dis. NOS, Peripheral vascular disease monitoring, Peripheral vascular disease NOS, Phlebitis NOS, Phlebitis of a superficial leg vein NOS, Polyarteritis nodosa, Portal vein thrombosis, Raynaud's disease, Raynaud's phenomenon, Raynaud's syndrome, Superficial vessel phlebitis and/or thrombophlebitis of leg, Telangiectasia, Varicose veins of legs, Varicose veins of the leg NOS, Varicose veins of the leg with eczema, Varicose veins of the leg with ulcer and eczema, Varicose veins of the legs

- Brain dysfunction

Cerebral palsy, Stroke, Demyelinating diseases of the central nervous system, Other cerebral infarction, Vascular dementia, Acute confusional state, Arnold - Chiari syndrome, Benign essential tremor, Benign intracranial hypertension, Brain injury NOS, Central demyelination of corpus callosum, Central pontine myelinosis, Central post-stroke pain, Cerebellar ataxia NOS, Cerebellar stroke syndrome, Cerebral aneurysm, nonruptured, Cerebral degeneration due to multifocal leukoencephalopathy, Cerebral palsy with spastic diplegia, Cerebrovascular disease, Chiari's malformation, CNS diseases (GMS), Cognitive decline, Congenital cerebral palsy, Congenital cerebral palsy NOS, CVA - cerebral artery occlusion, CVA - cerebrovascular accid due to intracerebral haemorrhage, CVA - Cerebrovascular accident unspecified, CVA unspecified, Dandy - Walker syndrome, Epilepsy, Epilepsy medication review, Epilepsy NOS, Epileptic seizures – tonic, CVA/stroke, TIA, Haematoma NOS, Hydrocephalus, Hydronephrosis, Left sided CVA, Mild cognitive impairment, Motor neurone disease, Multiple sclerosis, Multiple sclerosis of the spinal cord, Parkinson's disease, Progressive supranuclear palsy, Stroke and cerebrovascular accident unspecified, Stroke Monitoring, Stroke unspecified, Subarachnoid haemorrhage, Suspected stroke, Systemic sclerosis, Transient ischaemic attack, Traumatic brain injury, Traumatic subarachnoid haemorrhage, Unspecified encephalopathy.

- Bowel dysfunction

Change in bowel habit, Rectal symptoms, Stool contents abnormal, Stoma care, Has ileostomy, Altered bowel habit, Bile acid malabsorption syndrome, Bleeding PR, Blood in stool, Bowel dysfunction, Bowel obstruction, Change in bowel habit, Chronic constipation with overflow, Clostridium difficile infection, Colitis - ulcerative (GMS), Collagenous colitis, Colon polyp, Colonic fistula, Colonic polyp, Colonic Polyp, Colovesical fistula, Constipated, Constipation, Constipation – functional, Constipation NOS, Constipation symptom, Crohn's colitis, Crohn's disease, Crohn's disease of the small bowel NOS, Drug induced constipation, Faeces: mucous present, Hirschsprung's disease, Ileal stricture, Incontinent of faeces, Incontinent of faeces symptom, Inflammatory bowel disease, Irritable bowel – IBS, Irritable bowel syndrome, Irritable bowel syndrome with diarrhoea, Irritable colon - Irritable bowel syndrome, Loose stools, Regional enteritis - Crohn's disease, Small bowel obstruction NOS, Soiling symptom.

- Burning sensation

Burning feet, Burning feet syndrome, Burning pain

- Cancer

Adenocarcinoma NOS, Adenocarcinoma, metastatic, Adenocarcinomas, Carcinoma, metastatic, Epithelioid mesothelioma malignant, Leiomyosarcoma, Lymphoma, Malignant lymphoma large cell, cleaved, diffuse, Malignant melanoma, Mesothelioma malignant, Myeloma, Neoplasm metastatic, Non Hodgkins lymphoma, Pancreatic adenomas and carcinomas, Papillary carcinoma, Renal adenoma/carcinoma, Sarcoma, Spindle cell sarcoma, Squamous cell carcinoma, Squamous cell carcinoma metastatic, Transitional cell papillomas and carcinomas, Family history of malignant neoplasm of breast, Adenocarcinoma cervix uteri stage 3 (GMS), Adenocarcinoma of lung, B-cell chronic lymphocytic leukaemia, Breast carcinoma, Breast carcinoma left, Ca female breast, Carcinoma, Carcinoma bladder, Carcinoma breast, Carcinoma breast left had, Carcinoma in situ of breast, Carcinoma in situ of bronchus or lung, Carcinoma in situ of cervix uteri, Carcinoma in situ of oral cavity, Carcinoma in situ of prostate, Carcinoma in situ of tongue, Carcinoma in situ of upper lobe bronchus and lung, Carcinoma of rectum, Chronic lymphatic leukaemia, Chronic myeloid leukaemia, Colonic cancer, Diffuse large B-cell lymphoma, prostate cancer, Hepatocellular carcinoma, Hodgkin lymphoma, Lung cancer, Malig neop larynx, Malig neop of colon, Malig neop soft palate, Malignant lymphoma, Malignant melanoma of skin, Malignant neoplasm of anal canal, Malignant neoplasm of bronchus or lung, Malignant neoplasm of cervix uteri, Malignant neoplasm of cervix uteri, Malignant neoplasm of colon, Malignant neoplasm of connective and other soft tissue, Malignant neoplasm of connective and soft tissue of axilla, Malignant neoplasm of descending colon, Malignant neoplasm of female breast, Malignant neoplasm of female breast, Malignant neoplasm of floor of mouth, Malignant neoplasm of greater vestibular (Bartholin's) gland, Malignant neoplasm of labia minora, Malignant neoplasm of larynx, Malignant neoplasm of middle lobe, bronchus or lung, Malignant neoplasm of oesophagus, Malignant neoplasm of other site of cervix, Malignant neoplasm of ovary, Malignant neoplasm of pancreas, Malignant neoplasm of prostate, Malignant neoplasm of sigmoid colon, Malignant neoplasm of soft palate, Malignant neoplasm of stomach, Malignant neoplasm of subglottis, Malignant neoplasm of thyroid gland, Malignant neoplasm of tongue, Malignant neoplasm of tonsil, Malignant neoplasm of tonsillar fossa, Malignant neoplasm of upper lobe, bronchus or lung, Malignant neoplasm of urinary bladder, Multiple myeloma, Myelodysplasia, Oesophageal cancer, Prostate cancer care review, Rectal carcinoma, Renal malignant neoplasm, Suspected bladder cancer, Suspected lung cancer, Suspected malignancy.

- Chest discomfort

Chest discomfort, Chest lump, Chest pain, Chest pain, unspecified, Chest tightness, Chest injury, Intercostal myalgia, Intercostal neuropathy, Pleuritic pain, Retrosternal pain

- Chronic intractable pain

Chronic intractable pain, Other chronic pain, Myofascial pain syndrome, Polysymptomatic

- Chronic regional pain syndrome

Chronic regional pain syndrome, Complex regional pain syndrome, Complex regional pain syndrome type I, Sympathetic nerve dystrophy syndrome

- Consultation

Advice, Advice about treatment given, Advice to GP to start patient medication, Discussed with carer, Discussion, Discussion with colleague, DNA hospital appointment, Emergency appointment, Follow-up consultation, Fostering medical examination, Had a chat to patient, Had a discussion with patient, Home visit, Home visit elderly assessment, Home visit request by patient, Learning disabilities annual health assessment, Letter from consultant, Letter from specialist, Letter/report awaited, MED3 - doctor's statement, New patient consultation, New patient health check, New patient screen, New patient screen - problem identified, New patient screen admin, New patient screen admin, New patient screen done, New patient screen, NHS Health Check programme, Patient asked to come in, Patient given advice, Patient health questionnaire (PHQ-9) score, Patient medication advice, Patient non compliant with specific advice, Patient refuses hospital admit, Patient's condition deteriorating, Planned telephone contact, Telephone call to a patient, Telephone consultation, Telephone encounter, Telephone triage encounter

- Cough

Cough syncope, Haemoptysis, cough, Chesty cough, Chronic cough, Cough, Cough symptom, Coughing up phlegm, Dry cough, Nocturnal cough / wheeze, Persistent cough, Productive cough -clear sputum, Productive cough -green sputum, Productive cough, Productive cough-yellow sputum

- Cramping

Claudic.- intermittent, Claudication, Intermittent claudication, night cramps, hand cramps, leg cramps, cramps, cramping pain,

- Dental complaints

Dental health promotion, Wisdom tooth, Acute pericoronitis, Dental abscess, Dental caries, Dental infection, Dental symptoms, Gingival hyperplasia, Sensitive teeth dentine, Simple dental extraction, Simple extraction of tooth, Surgical removal of impacted wisdom tooth, Tooth symptoms, Toothache

- Diabetes

Hyperglycaemia, Impaired fasting glucose, Chronic painful diabetic neuropathy, Diabetes mellitus, Diabetic - poor control, Diabetic Charcot arthropathy, Diabetic nephropathy, Diabetic neuropathy, Diabetic on insulin, Diabetic polyneuropathy, Diabetic retinopathy screening, Insulin dependent diab mellit, Insulin treated Type 2 diabetes mellitus, Left diabetic foot – ulcerated, Type 1 diabetes mellitus, Type 1 diabetes mellitus with gastroparesis, Type 2 diabetes mellitus, Type 2 diabetes mellitus with exudative maculopathy, Type II diabetes mellitus

- Drug misuse

Drug addiction – opioids, Heroin addiction, Mental and behav dis due seds/hypntcs: withdrawal state, Mental and behav dis due to use opioids: dependence syndr, Analgesic abuse, Benzodiazepine dependence, Cocaine type drug dependence, Combined opioid with other drug dependence, Continuous opioid dependence, Diazepam dependence, Methadone dependence, Morphine dependence, Other drug psychoses, Other specified drug dependence, unspecified, Overdose of drug, Suspected drug abuse

- Endometriosis

Acute endometritis, Endometriosis, Endometriosis, Other endometriosis

- ENT complaint

Blocked ear, Difficulty in swallowing, Epistaxis, Acute infective otitis externa, Acute left otitis media, Acute otitis media with effusion, Acute pharyngitis, Acute pharyngitis, Acute rhinosinusitis, Acute right otitis media, Acute suppurative otitis media, Acute tonsillitis, Acute viral tonsillitis, Allergic rhinitis, Allergic rhinosinusitis, Barrett's oesophagus, Blocked nose, Buzzing in ear, post nasal drip, Calculus – salivary, Cholesteatoma of middle ear, Chronic otitis media with effusion, serous, Chronic rhinitis, Chronic rhinosinusitis, Chronic simple rhinitis, Chronic suppurative otitis media, Coryza – acute, Deafness, Difficulty swallowing solids, Dry mouth, Dysphagia, Ear drum perforation, Ear pain, Ear symptoms, Ear/nose/throat symptoms, Earache symptoms, Eustachian tube dysfunction, Glue ear, hearing problem, Has a sore throat, Has nose bleeds – epistaxis, Hearing difficulty, Hearing impairment, Hearing loss, High frequency deafness, Hoarse, Hoarseness symptom, Impacted cerumen (wax in ear), Inflamed throat, Labyrinthitis, Mallory - Weiss tear, Meniere's disease, Nasal congestion, Nasal symptoms, Nasal symptoms, Nasal turbinate hypertrophy, Nose bleed symptom, Nose cellulitis/abscess, Nose symptoms, Nutcracker oesophagus, foreign body in ear, wax in ear, Oesophageal dysmotility, Otalgia, Other otitis externa, Other vocal cord disease, Otitis externa, Otitis externa, Otitis media, Perennial rhinitis, Perforation of oesophagus, Pharyngitis - acute-as above, red throat with (GMS), Ramsey - Hunt syndrome, Rhinitis – acute, Rhinitis – chronic, Spasm of oesophagus, Stricture of oesophagus, Suppurative and unspecified otitis media, Swallowing symptoms, Throat symptom, Tinnitus, Tinnitus symptom, Tinnitus symptoms, Tongue symptoms, Tonsillectomy, Tonsillitis, Tympanic membrane perforation, Ulcer of oesophagus, Unilateral earache, Unspecified otalgia, Viral labyrinthitis, Viral sore throat, Voice hoarseness, Wax in ear

- Eye complaint

Adherent prepuce, Amaurosis fugax, Anisocoria - unequal pupil diameter, Bilateral cataracts, Blind In Right Eye, Blurred vision, Cataract, Corneal abrasion, Dry eye syndrome, Dry eyes, Dry senile macular degeneration, Ectropion, Eye pain, Eye symptoms, Glaucoma, Has a red eye, Homonymous hemianopia, Hordeolum externum ( stye ), Itchy eye symptom, Ocular hypertension, Ophthalmic migraine, Optic neuritis, Primary open-angle glaucoma, Raised intra-ocular pressure, Retinal detachment, Scleritis, Seen by optician, Sticky eye, Thyroid eye disease, Unspecified amblyopia, Unspecified conjunctivitis, Watery eyes

- Facial pain

Face ache, Facial pain, Facial nerve (VII), Bell's (facial) palsy, Facial swelling, Facial weakness, facial injury, Orofacial dyskinesia, Stickler syndrome

- Fever

Fever symptoms, Hot flushes, Menopausal flushing, Menopausal or female climacteric state, Rigor – symptom, Scarlet fever, Temperature symptoms

- Flu

Flu like illness, Influenza vaccination, Seasonal influenza vaccination

- Gastroenterology problems

Gamma glutaryl transferase raised, Hepatomegaly, Nausea, Nausea and vomiting, Right upper quadrant pain, Other gastritis, Acid reflux, Acute cholecystitis, Acute gastritis, Acute pancreatitis, Acute pancreatitis, Anal fissure, Anal fissure and fistula, Anal pain, Anal symptoms, Angiodysplasia of colon, Autoimmune hepatitis, Balloon gastrostomy feeding, Biliary colic, Campylobacter enteritis, Cholecystitis, Cholelithiasis, Cholelithiasis, Chronic anal fissure, Chronic cholecystitis, Chronic deafness, Chronic gastritis, Chronic liver disease, Chronic pancreatitis, Cirrhosis - non alcoholic, Cirrhosis and chronic liver disease, Cirrhosis of liver, Coeliac disease, Diarrhoea, Diarrhoea & vomiting, symptom, Diarrhoea symptom, Diarrhoea symptoms, Divertic disease/both sml+lge intestin with perforat+abscess, Diverticular abscess, Diverticular disease, Diverticulitis, Diverticulitis, Diverticulitis of the colon, Diverticulosis, Diverticulosis of the colon, Duodenal diseases, Duodenal ulcer, Duodenal ulcer, Duodenal ulcer, Duodenal ulcer, Duodenitis, Dyspepsia, Dyspepsia-long hx.Scoped in, Enteritis - presumed infectious origin, Exacerbation of ulcerative colitis, Fatty liver, Gallbladder calculus with acute cholecystitis, Gallbladder calculus without mention of cholecystitis, Gallstones, Gastric ulcer, Gastric ulcer, Gastritis and duodenitis, Gastritis unspecified, Gastro-oesophageal reflux, Gastroenteritis,Gastroenteritis - presumed infectious origin, Gastrointestinal symptoms, Gastrointestinal symptoms, Gastroscopy abnormal, Gastroscopy NEC, Gilbert's syndrome, GIT symptom changes, Gluten intolerance, colitis, peptic ulcer, Haematemesis, Heartburn, Heartburn symptom, Helicobacter eradication therapy, Helicobacter gastritis, Helicobacter pylori breath test, Helicobacter pylori gastrointestinal tract infection, Helicobacter pylori test positive, Helicobacter serology positive, History of acute pancreatitis, Hyperemesis gravidarum, Hyperemesis of pregnancy, Hyperhidrosis symptom, Indigestion, Indigestion symptoms, Laryngopharyngeal reflux, Liver cyst, Microscopic colitis, Nausea, Nausea symptoms, Non-alcoholic fatty liver, Nonalcoholic steatohepatitis, Oesophageal reflux, Oesophagitis, Other liver disorders, Pancreatitis, Percutaneous endoscopic gastrostomy feeding, Pernicious anaemia, Post cholecystectomy bile leakage, Reflux oesophagitis, Reflux oesophagitis (GMS), Reflux oesophagitis abdomen, Right upper quadrant pain, Seasickness, Suspected gallstones, Viral gastroenteritis, Wilson's disease

- General aches and pains

Allodynia, General aches and pains, Non cardiac chest pain, Pain, generalized, Aches and pains, generally unwell (GMS), Aching leg syndrome, Aching muscles, Aching pain, a pain, an ache, a pain, Chronic pain, Chronic pain review, Constant pain, Dyspareunia, Generalised pain [symptom], dyspareunia, Mastitis, Mastitis - non puerperal, Mastodynia - pain in breast, Muscle injury / strain, Muscle pain, Muscle sprain, Muscle strain, Night sweats, Night terrors, in pain, Pain, Pain and symptom management, Pain control, Pain in testicle, Pain management, Pain management (specialty), Pain relief, Pain relief by medication, Pain, generalized, Pain, generalized, Painful scar, Painful swallowing, Painful tongue, Persistent mastalgia, Premenstrual tension syndrome, Rectal bleeding, Rectus muscle sprain, Shaking, Soft tissue disorders

- Groin discomfort

Groin lump, Groin pain, Groin, Cellulitis and abscess of groin, Groin Pain, Groin sprain, Groin strain (GMS)

- Gynecological or reproductive issues

Fibroid uterus, Postnatal care, Vaginal prolapse, General contraceptive advice and counselling, Removal of coil, Removal of intrauterine contraceptive device, Sterilisation, Other specified, menopausal and perimenopausal disorders, Abnormal vaginal bleeding, unspecified, Bacterial vaginosis, p.v. bleeding, Candidal vulvovaginitis, Cervical neoplasia screen, Cervical neoplasia screening, Cervical nerve root injury - C7, Cervical smear due, Cervical smear overdue, Cervical smear screen, Cervical smear taken, Cervical spinal stenosis, Cold coagulation of lesion of cervix, Combined oral contraceptive, Contraception, Cyst of Bartholin's gland, Cystocele with second degree uterine prolapse, Cystocele without uterine prolapse, Delayed menstruation, Delayed period, Depo-provera injection given, Depot contraception, Divarication of recti, Dysfunctional uterine bleeding, Emergency contraception, Endometrial polyp, Fertility problem, Fibroids, First degree perineal tear during delivery, Full post-natal examination, General contraceptive advice, Genital prolapse, Gynaecological history, miscarriage, painful periods, Heavy periods, High vaginal swab taken, Hot flushes – menopausal, Hydrosalpinx, Hysteroscopy NEC, Infertility investigation -fem, Infertility problem, Intermenstrual bleeding, Intermenstrual bleeding – irregular, Introduction of Mirena coil, Irregular menstrual cycle, Maternal P/N 6 week exam, Menopausal and postmenopausal disorders, Menopausal symptoms, Menopausal symptoms, Menopause, Menopause symptoms present, Menorrhagia, Menstruation disorder, Menstruation disorders, Miscarriage, Missed miscarriage, Missed period, Mittelschmerz - ovulation pain, Obstetric history, Oral contraception, Oral contraception, Oral contraceptive, Oral contraceptive repeat, Ovarian cyst, Ovarian cysts, Patient currently pregnant, Patient pregnant, Perimenopausal menorrhagia, Period disorders, Period pains, Phantom pregnancy, Polycystic ovarian syndrome, Post natal care, Postcoital bleeding, Postmenopausal atrophic vaginitis, Postmenopausal bleeding, Postnatal care, Postnatal exam. – maternal, Postnatal examination normal, Pregnancy complications, Premature menopause, Progestogen only oral contraceptive, Rectocele, Rectocele without uterine prolapse, Replacement of Mirena coil, Requests pregnancy termination, Second degree perineal tear during delivery, Secondary amenorrhoea, Secondary dysmenorrhoea, Separation of vulval adhesions, Sexually transmitted diseases, Termination of pregnancy, Therapeutic endoscopic operations on uterus, Third degree perineal tear during delivery, Threatened abortion, Thrush, Total abdominal hysterectomy, Total abdominal hysterectomy with conservation of ovaries, Trying to conceive, Unprotected intercourse, Unwanted pregnancy, Urine dipstick test, Urine pregnancy test positive, Uterine leiomyoma – fibroids, Uterovaginal prolapse, incomplete, Uterovaginal prolapse, unspecified, Vaginal discharge, Vaginal discharge symptom, Vaginal discomfort, Vaginal dryness, Vaginal hysterectomy, Vaginal irritation, Vaginal pain, Vaginal thrush, Vaginal wall prolapse without uterine prolapse, Vaginitis and vulvovaginitis, Vaginitis unspecified, Vulva sore, Vulval irritation, Vulval pain, Vulval sores, Vulvectomy, Vulvitis unspecified, Vulvodynia, Wishes to postpone menstruatn.

- Headache

Headache, Pain in head, Tension type headache, Cervicogenic headache, Chronic headache disorder, Tension type headache, a headache, Chronic paroxysmal hemicrania, Classical migraine, Cluster headache, Frontal headache, migraine, trigeminal neuralgia, Headache, Headache - post traumatic, Medication overuse headache, Migraine, Migraine – menstrual, Migraine, Migraine with aura, Muscular headache, Occipital headache, Other forms of migraine, Other specified trigeminal neuralgia, Paroxysmal hemicrania, Sinus headache, Temporal arteritis, Temporal headache, Tension headache

- Heart condition

Bumping of heart, Bradycardia, unspecified, Cardiac pacemaker in situ, Bradycardia, unspecified, Other hypertrophic cardiomyopathy, Acute coronary syndrome, Acute myocardial infarction, Acute non-ST segment elevation myocardial infarction, Acute pericarditis, Acute ST segment elevation myocardial infarction, Angina pectoris, Angina pectoris, Aortic regurgitation alone, cause unspecified, Aortic stenosis, Aortic valve disorders, Atrial fibrillation, Atrial fibrillation and flutter, Atrial flutter, Atrial septal defect, Cardiac arrhythmias, Cardiomyopathy, Cardiovascular symptoms, Congestive heart failure, Constrictive pericarditis, Coronary artery disease, First degree atrioventricular block, Heart failure, IHD - Ischaemic heart disease, Impaired left ventricular function, Ischaemic heart disease, Left ventricular diastolic dysfunction, Left ventricular failure, Left ventricular hypertrophy, Left ventricular systolic dysfunction, MI - acute myocardial infarct, MI - acute myocardial infarction, Myocardial infarct INPAT AINTREE (GMS), Palpitations, Paroxysmal atrial fibrillation, Primary dilated cardiomyopathy, Primary prevention of ischaemic heart disease, QRISK2 cardiovascular disease 10 year risk score, Refractory angina, Sinus tachycardia, Stable angina, Suspected ischaemic heart disease, Valvular heart disease

- Hormone replacement

Hormone replace monitor admin, Hormone replacement therapy, Hormone Replacement Therapy ongoing treatment

- Hypertension

Palpitations, Raised blood pressure read, Raised blood pressure reading, Examination of blood pressure, Benign essential hypertension, Essential hypertension, Essential hypertension, hypertension, Hypertension, Hypertension (GMS), Hypertension annual review, Hypertension medication review, Hypertension monitored, Hypertension monitoring, Hypertension, Hypertensive disease, Hypertensive disease, blood pressure, blood pressure reading, BP borderline raised, Postural hypotension, White coat hypertension

- Infection

Gangrene, Abscess of axilla, Abscess of buttock, Cellulitis of breast, Perineal abscess, Abscess, Abscess of Bartholin's gland, Abscess of jaw, Abscess of labia, Abscess of vulva, Acute bacterial tonsillitis, Acute bilateral otitis media, Acute conjunctivitis, Acute follicular tonsillitis, Acute laryngitis, Allergic conjunctivitis, Allergy, unspecified, Antimalarial drug prophylaxis, Biliary sepsis, Blepharo conjunctivitis, Boil of axilla, Boil of vulva, Boils of multiple sites, Breast abscess, Breast infection, Candidal intertrigo, Candidiasis, Cervical discitis, Chest infection, Chest infection - pnemonia due to unspecified organism, Chest infection - unspecified bronchitis, Chest infection, Community acquired pneumonia, Conjunctivitis, Cutaneous cellulitis, Drainage of abscess, Drainage of perianal abscess, Epididymo-orchitis, Epidural intraspinal abscess, Eye infection, Flea bite, Fungal infection of skin, Fungal nail infection, Furuncle – boil, Gangrene of finger, Genital herpes unspecified, Genital warts, chronic ear infection, poliomyelitis, viral illness, Hand, foot and mouth disease, Having rigors, Hepatitis C, Herpes simplex, Herpes zoster, Herpes zoster ophthalmicus, HIV positive, Impetigo, Incision and drainage of abscess, Infected eczema, Infected insect bite, Infected joint prosthesis, Infected nailfold, Infected sebaceous cyst, Infected skin ulcer, Infected varicose ulcer, Infection ear, Infection finger, Infection foot, Infection toe, Infective endocarditis in diseases EC, Infective otitis externa, Ingrowing nail with infection, Insect bite, Insect Bites, Insect bites - non venomous, Intraspinal abscess, Ischaemic leg ulcer, Ischiorectal abscess, Local infection skin/subcut tissue, Major aphthous ulceration, Meticillin resistant staphylococcus aureus, Mouth ulcer, Mumps, Nail infection, Nasal infection, Nasopalatine cyst, Non-healing leg ulcer, ankle ulcer, infected toe, Right foot ulcer, ulcer on tongue, Wound infected, Onychomycosis, Oral aphthae, Oral candidiasis, Oral cavity, salivary gland and jaw diseases, Oral thrush, Pancreatic cyst, Paronychia of finger, Paronychia of toe, Penile candidiasis (thrush), Perianal abscess, Perianal candidiasis, Peritonitis, Peritonsillar abscess – quinsy, Pilonidal sinus/cyst, Pilonidal sinus/cyst, Pneumococcal meningitis, Pneumonia or influenza, Post-traumatic wound infection, Postoperative infection, Postoperative stitch abscess, Postoperative wound abscess, Postoperative wound infection, unspecified, Psoas abscess, Sebaceous cyst, Sebaceous cyst – wen, Skin abscess, Skin and subcutaneous tissue infections, Splenic cyst, Syringomyelia, Syringomyelia/syringobulb, Throat infection – pharyngitis, Throat infection – tonsillitis, Tinea, Tinea corporis, Tinea cruris, Tinea pedis, Traumatic leg ulcer, Ulcer of skin, Viral infection, Whitlow, cold sore

- Ingrowing toe nail

Ingrowing great toe nail

- Lump on body

Axillary lump, Local superficial swelling, mass or lump, Local superficial swelling, mass or lump, Lump on hand, Lump on leg, Lump on shin, Lump stomach, Lump, localized and superficial, Swelling, mass or lump in neck, Breast lump present, Breast lump symptom, Dercum's disease, Dermoid cyst, External thrombosed haemorrhoids, Feeling of lump in throat, Haemorrhoids, Hernia – incisional, Hernia of abdominal cavity, Incisional hernia, Indirect inguinal hernia, Inguinal hernia, Internal haemorrhoids, simple, Left inguinal hernia, Lump in breast, Lump on neck, a lump, Piles – haemorrhoids, Rupture of Baker's cyst – knee, Sarcoidosis

- Lupus

Lupus erythematosus, Lupus nephritis, Systemic lupus erythematosis (GMS), Systemic lupus erythematosus

- Lymphadenopathy

Lymph node enlargement, Lymphadenopathy, Other nonspecific lymphadenitis, Acute lymphadenitis, Congenital lymphoedema, Follicular lymphoma, Follicular non-Hodgkin's lymphoma, Kikuchi disease, Milroy's disease, Non - Hodgkin's lymphoma, lymphadenopathy, cervical lymphadenopathy, Other lymphoedema

- Malaise

Malaise, Malaise and fatigue, Feels unwell, Malaise/lethargy, Sickness notification-of GP

- Male genitourinary tract

BPH - Benign prostatic hypertrophy, Chronic prostatitis, Epididymal cyst, Epididymitis, Erectile dysfunction, Haematospermia, Hydrocele, Impotence, Oligoasthenozoospermia, Orchitis, Penile disorders, Peyronie's disease, Phimosis, Premature ejaculation, Seen by urologist, Seminoma of testis

- Medication requested

Analgesics requested, Hospital prescription, Lost prescription, Medication requested, Patient requested treatment

- Medication review

Anticoagulant monitoring, Buprenorphine maintenance therapy, Initial post discharge review, Medicals/reports, Medication change to generic, Medication changed, Medication dispensed in error, Medication error, Medication recommenced, Medication started, New medication added, New medication commenced, On repeat dispensing system, Ongoing review, Other medication management, Patient reviewed, Pct Anti-Coag Monitoring, Pill check, Polypharmacy, Polypharmacy medication review, Prescription collected by patient, Prescription given no examination of patient, Prescription issued for patient on holiday, Previously Active Medications imported via GP2GP, Warfarin monitoring, epilepsy medication review, hypertension med review, med review no surgery, med review with out patient, med review with patient, med review medical notes, med review done, med review by pharmacist, med review by Dr, med review done, med review.

- Memory

Unspecified dementia, Alzheimer's dementia unspec, Dementia in Alzheimer's disease, Alzheimer's disease, Carer of person with dementia, Memory assessment, Memory disturbance, Memory loss symptom, Mild memory disturbance

- Mental health

Irritability and anger, Work stress, Behavioural problems, Reactive depression, Agoraphobia, Anxiety, Anxiety reaction, Anxiety state, Attention deficit hyperactivity disorder, Bipolar affective disorder, Borderline schizophrenia, Chronic pain personality syndrome, Cyclothymia, Cyclothymic personality, Deliberate drug overdose / other poisoning, Delusional disorder, Depression NOS, Depressive disorder, Depressive episode, Depressive episode, unspecified, Dissocial personality disorder, Eating disorder, unspecified, Eating disorders, Emotionally unstable personality disorder, Endogenous depression without psychotic symptoms, Generalized anxiety disorder, Grief reaction, Induced psychotic disorder, Intentional self harm by other specified means, Mixed anxiety and depressive disorder, Moderate depressive episode, Neurotic depression, Obsessive - compulsive disorder, Panic disorder+agoraphobia, Paranoia, Post - traumatic stress disorder, Psychosis, Recurrent depressive disorder, Recurrent depressive disorder, currently in remission, Recurrent depressive disorder, unspecified, Schizoaffective disorder, unspecified, Severe depressive episode with psychotic symptoms, Severe depressive episode without psychotic symptoms, Trichotillomania, Acute reaction to stress, Acute stress reaction, Agitated, Agoraphobia with panic attacks, Agreeing on mental health care plan, Anger management counselling, Anger reaction, Anorexia nervosa, Antisocial or sociopathic personality disorder, Anxiety state, Anxiety state, Anxiety state unspecified, Anxiety states, Anxiety with depression, Anxiousness, Appetite loss – anorexia, Attempted suicide, Behaviour disorder, Bereavement, Bereavement reaction, Bipolar affective disorder, Borderline personality disorder, feeling depressed, Cause of overdose – deliberate, Chronic anxiety, Chronic depression, Confusion, Death of father, Death of mother, Death of spouse, Depressed, Depressed mood, Depression, Depression annual review, Depression interim review, Depression, Depression stable,mother, Depressive disorder, Depressive symptoms, Domestic stress, Emotional upset, Emotionally unstable personality, Endogenous depression, Family bereavement, Family problems, Fear of flying, Feeling stressed, Flashbacks, Forgetful, Generalised anxiety disorder, Grief reaction, deliberate self harm, depression, psychiatric disorder, Irritable, Life crisis, Low mood, Medication counselling, Mental health review, Mini mental state score, Mixed anxiety and depressive disorder, Mixed bipolar affective disorder, Moderate depression, Neurotic (reactive) depression, Neurotic depression reactive type, anxious, Other personality disorders, Other post-traumatic stress disorder, Panic attack, Paranoid psychosis, Paranoid schizophrenia, Personality disorder, Personality disorders, Postnatal depression, Reactive depression (GMS), Recurrent anxiety, Recurrent depression, Restless, Schizo-affective schizophrenia, SCHIZOPHRENIA, Schizophrenia, Schizophrenic disorders, Schizophrenic psychoses, Seasonal affective disorder, Single major depressive episode, moderate, Stress at home, Stress at work, Stress counselling, Stress related problem, Suicidal ideation, Symptoms of depression, Tearful, Visual disturbances, Worried

- Multiple symptoms

Multiple symptoms

- Musculoskeletal pain

Musculoskeletal chest pain, Musculoskeletal pain, Musculoskeletal symptoms, Soft tissue injuries, Acromioclavicular joint, Anterior cruciate ligament, Arm, Gastrocnemius, Gluteus, Gluteus medius, Greater trochanter, Hip joint, Ischial tuberosity, Knee joint, Lip, Nose, Patellofemoral joint, Posterior horn of medial meniscus, Prepatellar bursa, Pubic symphysis, Rib cage, Rotator cuff, Sacroiliac joint, Scapular region, Shoulder joint, Soft tissue, Sole of foot, Third metatarsal, Trapezius, Trochanter of femur, unspecified, Ulnar collateral ligament, Foot problem, Problem knee, Toe problem, Dependence on wheelchair, Unspecified limb or other problem, Arthrosis, Dystonia, unspecified, Inflammatory myopathy, not elsewhere classified, Injuries involving multiple body regions, Injuries to the ankle and foot, Injuries to the knee and lower leg, Mixed connective tissue disease, Other bursitis of knee, Other disorders of patella, Other osteonecrosis, Unspecified injury of shoulder and upper arm, Unspecified multiple injuries, ankle, Clavicle, Femur, Abnormal gait, Ache in joint, Achilles bursitis, Achilles tendinitis, Acquired ankle or foot deformity, Acquired deformity, Acromio-Clavicular Dislocation, Acute exacerbation of gout, Acute meniscal tear, lateral, Acute meniscal tear, lateral, bucket handle tear, Acute meniscal tear, medial, Acute meniscal tear, medial, posterior horn, Adhesive capsulitis of the shoulder, Amputation, Amputation above knee, Amputation below knee, Amputation great toe, Amputation hallux, Amputation of leg, Amputation of leg, Amputation of toe, Amputation through knee, Ankle and foot sprain, Ankle joint pain, Ankle pain, Ankle pathological dislocation, Ankle sprain, Ankle stiff, Ankle swelling, ,Ankle/foot joint pain, Ankle/foot joint pain long, Ankylosing spondylitis, Ankylosing spondylitis the, Aquired cavus foot deformity, Arm bruise, Arm pain, Arthralgia, Arthralgia - ankle/foot, Arthralgia - lower leg, Arthralgia – shoulder, Arthralgia - site unspecified, Arthralgia NOS, Arthralgia of 1st MTP joint, Arthralgia of acromioclavicular joint, Arthralgia of hip, Arthralgia of IP joint of toe, Arthralgia of knee, Arthralgia of multiple joints, Arthralgia of sacro-iliac joint, Arthralgia of shoulder, Arthralgia of sternoclavicular joint, Arthralgia of the ankle and foot, Arthralgia of the hand, Arthralgia of the lower leg, Arthralgia of the pelvic region and thigh, Arthralgia of the shoulder region, Arthralgia of unspecified site, Arthralgia of wrist, Avascular bone necrosis, Avascular necrosis of bone, Avascular necrosis of other bone, Avascular necrosis of the head of femur, Avascular necrosis of the head of humerus, Avascular necrosis-bone, Axial spondyloarthritis, Axillary nerve injury, Axillary pain, Bilateral dysplastic hip, Brachial (cervical) neuritis, Brachial neuritis - bilaterastretch testing and (GMS), Brachial neuritis - rom neck nad shoulder nad (GMS), Breast soreness, Breast tenderness, Bunion, Bursitis NOS, Bursitis of hip, Bursitis of the knee, stiffness, Calcaneal spur, Calcific tendinitis, Calcifying tendinitis of the shoulder, Calf injury, Carpal tunnel syndrome, Cervical cord injury without evidence of spinal bone injury, Cervical disc, Cervical disc degeneration, Cervical disc disorder with radiculopathy, Cervical disc displacement, Cervical disc displacement without myelopathy, Cervical disc prolapse with myelopathy, Cervical disc prolapse with radiculopathy, Cervical radiculitis, Cervical rib syndrome, Chondrocalcinosis, Claw hand – acquired, Cleidocranial dysostosis, Closed flail chest, Clubfoot, Complete division extensor tendon hand, Complete tear, knee, anterior cruciate ligament, Congenital hammer toe, Congenital talipes equinovarus, Contusion chest wall, Contusion knee, Costochondral joint syndrome, Costochondritis, Costochondritis, CTS - Carpal tunnel syndrome, Cubital tunnel syndrome, de Quervain's disease, De Quervain's disease, Developmental dysplasia of the hip, DHS - Dynamic hip screw primary fixation of neck of femur, Diffuse idiopathic skeletal hyperostosis, Disability, Disabled, Disc prolapse with radiculopathy, Dupuytren's contracture, Dupuytren's disease of palm, Dynamic hip screw primary fixation of neck of femur, Dysplastic hip, Dystonia, unspecified, Effusion of knee, Ehlers-Danlos syndrome, Ehlers-Danlos syndrome type III, Emery-Dreifuss muscular dystrophy, Endocr./nutrit/metabol.disease, Extensor tenosynovitis of wrist, Facioscapulohumeral muscular dystrophy, Feet deformities, Female pelvic inflammatory diseases, Femoroacetabular impingement, Finger trigger, Forestier's disease, Gluteal tendinitis, gout, knee problem kissing, Hallux rigidus – acquired, Hallux valgus – acquired, Hallux valgus osteotomy, Hamstring sprain, Hand rheumatism, Heberdens' nodes, Hemiplegia, Hereditary spastic paraplegia, Hip # - closed reduct. (GMS), Hip DXA scan result osteopenic, Hip girdle aches, Hip prosthesis loose, Hypophosphataemia, Iliotibial band syndrome, Klippel-Feil syndrome, Knee gives way, Knee joint effusion, Kyphoscoliosis-acquired, Kyphoscoliosis/scoliosis, Lateral epicondylitis, Lateral epicondylitis – elbow, Lateral epicondylitis of the elbow, Left sided weakness, Medial epicondylitis of the elbow, Medial meniscus derangement, Meniscus derangement, Meniscus derangement NOS (GMS), Metatarsalgia, Mobility, Mobility poor, Moderate frailty, Multiple epiphyseal dysplasia, Multiple joint pain, Multiple joint pain,but right, Multiple joint pain, multiple joint pains, Multiple stiff joints, Muscular dystrophy, Musculoskelet/connectiv tissue (GMS), Musculoskelet/connectiv tissue. (GMS), Musculoskeletal and connective tissue diseases, Musculoskeletal and connective tissue diseases, Musculoskeletal pain – joints, Musculoskeletal pain (GMS), Musculoskeletal pain, Myalgia or myositis, Myalgia unspecified, Myalgia/myositis, Myalgic encephalomyelitis, Myalgic, Encephalomyelitis, Myopathy or muscular dystrophy, Myositis unspecified, Myotonia congenita (Thomsen's disease), Neck disorder NOS (GMS), Neck pain, Neck pain, Neck sprain, Neck sprain, Neck sprain, unspecified, joint movement painful, joint swelling, limping gait, muscle tone spastic, shoulder joint abn, Old anterior cruciate ligament disruption, Old tear of posterior horn of medial meniscus, Olecranon bursitis, Ollier's disease, Osteitis deformans - Paget's, Osteochondritis dissecans, Osteochondritis, Osteochondritis of knee, Osteomyelitis NOS upper tibia, Osteopenia (GMS), Osteopenia L-spine on Dexa (GMS), Osteoradionecrosis of jaw, Other acute meniscus tear, Other and unspecified kyphosis, Other ankle injury, Other chest wall injuries, Other elbow injuries, Other finger injuries, Other finger injuries, unspecified, Other foot injury, Other hip injuries, Other joint symptoms, Other knee injury, Other knee, leg, ankle and foot injuries, Other leg injury, Other limb-girdle muscular dystrophy, Other lip injuries, Other neck injuries, Other nose injuries, Other peripheral enthesopathies, Other shoulder injuries, Other symptoms – shoulder, Other tenosynovitis of hand or wrist, Other tenosynovitis of the hand, Other tenosynovitis of the wrist, Other thigh injuries, Other valgus foot deformities, Other wrist injuries, Paget's disease, Paget's disease of bone, PAIN FOOT, PAIN HIP, Pain Im Multiple Joints, Pain in arm, Pain in buttock, Pain in cervical spine, Pain in coccyx, Pain in elbow, Pain in eye, Pain in joint, Pain in joint – arthralgia, Pain in left leg, Pain in leg, Pain in limb, Pain in limb multiple (GMS), Pain in lower limb, Pain in lumbar spine, Pain in penis, Pain In Right Arm, Pain In Right Leg, Pain in thoracic spine, Pain in upper limb, Pain in wrist, Painful arc syndr – shoulder, Painful arc syndrome, Painful Elbow, Painful Right Knee, Partial tear, knee, anterior cruciate ligament, Patellar tendinitis, Patellofemoral disorder, Patellofemoral maltracking, Perforated diverticulum unspecified, Pes planus – acquired, Phobic disorder, Piriformis syndrome, Polycythaemia vera, Polymyalgia, Polymyalgia rheumatica, Polymyositis, PREPATELLAR BURSA, Prepatellar bursitis, Psoas tendinitis, Pubic symphysis separation, Radial styloid tenosynovitis, Reflex sympathetic dystrophy, Revision repair of rotator cuff, Rib pain, Rib sprain, Rib sprain, Rib sprain unspecified, Right hemiparesis, Rotator cuff shoulder syndrome and allied disorders, Rotator cuff sprain, Rotator cuff syndrome, Rotator cuff syndrome unspecify, Rotator cuff syndrome, unspecified, Rupture Achilles tendon, Rupture quadriceps tendon, Rupture supraspinatus tendon, Sacroiliac disorder, Sacroiliac ligament sprain, Sacroiliac sprain, Sacroiliac sprain rt with, Sacroiliac strain, Sacroiliitis, Seen in musculoskeletal clinic, Severe frailty, Shoulder syndrome, Shoulder tendonitis, Spasm of muscle, Specific disability rehab, Staghorn calculus, Subacromial impingement, Superficial injury chest wall NOS, without major open wound, Superficial injury of foot, Supraspinatus syndrome, Supraspinatus tendinitis, Supraspinatus tendonitis, Symphysis pubis separation, Synovitis and tenosynovitis, Synovitis of knee, Synovitis or tenosynovitis, Synovitis/tenosyn.- wrist, Temporomandibular joint disord, Temporomandibular joint disorder, Temporomandibular joint disorders, Temporomandibular joint-pain-dysfunction syndrome, Tendinitis, Tendon injury – hand, Tendon injury to hand, Tendon rupture, Tendonitis, Tenodesis, Tibialis posterior tendinitis, Torticollis, Trigger finger – acquired, Trigger thumb, Trochanteric bursitis, Trochanteric tendinitis, Unilateral dysplastic hip, Unilateral leg oedema, Wrist joint pain, Wrist pain, Wrist sprain, Wry neck, Wry neck symptom, Wry neck/torticollis, Cervical spond.- no, Cervical spond.- no myelopathy. **Cervical Spondylosis**: Cervical spond.with, Cervical spond.with myelopathy, Cervical spondylosis, CERVICAL SPONDYLOSIS, Cervical spondylosis (GMS), Cervical spondylosis with myelopathy, Cervical spondylosis with radiculopathy, Cervical spondylosis with vascular compression, Cervical spondylosis without myelopathy, Multiple-level cervical spondylosis without myelopathy, O.A.Cervical. **Mechanical Pain** - Gait abnormality, Jaw pain, Titubation, Genitofemoral nerve, Reduced mobility, Anterior chest wall pain, Anterior dislocation of shoulder, Anterior knee pain, Anterior shin splints, Atypical chest pain, Bone pain, a neck symptom, pain in big toe, renal pain, pelvic pain, Calf pain, Central chest pain, Cervical myelopathy, Cervicalgia, Cervicalgia - pain in neck, Chest pain, Chest pain, Chest wall pain, Chondromalacia patellae, Closed dislocation cervical spine, Closed traumatic dislocation of shoulder, Coccyx sprain, Congenital hip dysplasia, Difficulty in walking, Dislocation of elbow, Dislocation of finger or thumb not otherwise specified, Dislocation of hip, Dislocation of knee, Dislocation of shoulder, Dislocation of thumb, Dislocation or subluxation of knee, Dislocation or subluxation of shoulder, Elbow pain, Finger injury, Finger pain, Flank pain, Flat foot, Flexion deformity of finger, Foot drop, Foot pain, Foot sprain, Frozen shoulder, Full thickness rotator cuff tear, Golfer's elbow, dislocated shoulder, knee problem, significant knee disorder, Hand joint pain, Hand joint stiff, Hand operation, Hand pain, Heel pain, Hip joint pain, Hip joint pain (Left), Hip pain, Housemaids' knee, Hypermobility syndrome, Impingement syndrome of shoulder, Joint disorder of shoulder region, Joint disorders, Knee – dislocated, Knee joint pain, Knee joint pain Both, Knee joint pain both knees, Knee joint pain both, full, Knee joint pain, lt side ? OA, Knee pain, Knee sprain, Knee sprain, Left flank pain, Left iliac fossa pain, Leg pain, Locking knee, Loose body in elbow joint, Loose body in knee, MSG:Ankle pain, Proctalgia fugax, Rectal pain, Rectal prolapse, Recurrent joint dislocation, of the shoulder region, Release of contracture of shoulder joint, Restless legs syndrome, Right flank pain, Shoulder joint pain, Shoulder pain, Shoulder pain (GMS), Shoulder sprain, Shoulder sprain (GMS), Shoulder sprain, Shoulder strain, Sore bottom, Sore gums, Sore lip, Sore mouth, Sore mouth – symptom, Sore Neck, Sore throat, Sore throat symptom, Sprain of knee and leg, Sprain of medial collateral ligament of knee, Sprain of shoulder and upper arm, Sprain of wrist and hand, Sprain shoulder/upper arm, Sprain, ankle joint, lateral, Sprain, quadriceps tendon, Sprain, tendocalcaneus (Achilles tendon), Sprains and strains, Sprains and strains of joints and adjacent muscles, Stiff neck, Stiff neck symptom, Symptom: ankle/foot, Symptom: chest wall, Tennis elbow, Tennis elbow – epicondylitis, Testicular hypogonadism, Testicular lump, Testicular pain, Testicular swelling, Thigh pain, Throat pain, Throat soreness, Thumb pain, Toe pain, Unstable ankle, Weakness of arm, Weakness of leg, Weakness symptoms. **Fibromyalgia**: Fibromyalgia, Query Fibromyalgia. **Bone fracture**: Osteochondral, Rehabilitation following fracture, Fract of other and unspec parts of lumbar spine & pelvis, Fracture of other parts of shoulder and upper arm, Fracture of shoulder and upper arm, unspecified, Fractures of other skull and facial bones, Arm fracture, Closed Colles' fracture, Closed fracture ankle, bimalleolar, Closed fracture ankle, lateral malleolus, Closed fracture ankle, trimalleolar, Closed fracture ankle, trimalleolar, low fibular fracture, Closed fracture ankle, unspecified, Closed fracture cervical vertebra, burst, Closed fracture cervical vertebra, transverse process, Closed fracture distal phalanx, toe, Closed fracture distal radius, extra-articular, other type, Closed fracture distal tibia, Closed fracture distal tibia, extra-articular, Closed fracture finger proximal phalanx, base, Closed fracture lumbar vertebra, Closed fracture lumbar vertebra, wedge, Closed fracture metatarsal, Closed fracture metatarsal base, Closed fracture metatarsal shaft, Closed fracture multiple ribs, Closed fracture navicular, Closed fracture of calcaneus, Closed fracture of cervical spine, Closed fracture of cervical spine - no spinal cord lesion, Closed fracture of clavicle, Closed fracture of distal fibula, Closed fracture of elbow, unspecified part, Closed fracture of femur, intertrochanteric, Closed fracture of foot, Closed fracture of great toe, Closed fracture of lumbar spine - no spinal cord lesion, Closed fracture of neck of femur NOS, Closed fracture of pelvis NOS, Closed fracture of proximal humerus, anatomical neck, Closed fracture of proximal humerus, unspecified part, Closed fracture of radius and ulna, lower end, Closed fracture of seventh cervical vertebra, Closed fracture of spine, unspecified, Closed fracture of the distal humerus, Closed fracture of the distal radius unspecified, Closed fracture of the patella, Closed fracture of the proximal humerus, Closed fracture of the proximal tibia, Closed fracture of tibia and fibula, proximal, Closed fracture olecranon, extra-articular, Closed fracture olecranon, intra-articular, Closed fracture pelvis, multiple pubic rami – stable, Closed fracture pelvis, single pubic ramus, Closed fracture proximal humerus, greater tuberosity, Closed fracture proximal phalanx, toe, Closed fracture radius and ulna, distal, Closed fracture radius, head, Closed fracture radius, neck, Closed fracture rib, Closed fracture shaft of tibia, Closed fracture thoracic vertebra, Closed fracture thoracic vertebra, spondylolysis, Closed fracture thoracic vertebra, wedge, Closed fracture triquetral, Closed fracture-dislocation of pelvis, Closed fracture-dislocation shoulder, Closed fracture-dislocation, ankle joint, Closed fracture-dislocation, hip joint, Closed fracture-dislocation, knee joint, Closed fracture-dislocation, tarsometatarsal joint, Closed reduction of dislocation of patella, Closed reduction of fracture of shoulder, Congenital dislocation and subluxation of the hip, Congenital dislocation of hip, Elbow fracture – closed, Finger fracture, Fracture NOS, Fracture of acetabulum, Fracture of ankle, Fracture of ankle, NOS, Fracture of bones NOS, Fracture of calcaneus, Fracture of clavicle, Fracture of coccyx, Fracture of femur, NOS, Fracture of fibula alone, Fracture of great toe, Fracture of humerus, Fracture of humerus NOS, Fracture of lateral malleolus, Fracture of lower end of humerus, Fracture of lower end of radius, Fracture of lower leg, part unspecified, Fracture of lower limb, Fracture of lumbar vertebra, Fracture of mandible, closed, Fracture of metacarpal bone, Fracture of metatarsal bone, Fracture of nasal bones, Fracture of neck of femur, Fracture of one or more phalanges of foot, Fracture of patella, Fracture of radius AND ulna, Fracture of radius NOS, Fracture of rib, Fracture of sacrum, Fracture of scaphoid, Fracture of shaft of tibia, Fracture of spine without mention of spinal cord injury, Fracture of spine without mention of spinal cord lesion NOS, Fracture of sternum, Fracture of thoracic vertebra, Fracture of thumb, Fracture of tibia, Fracture of tibia and fibula, Fracture of tibia AND fibula, Fracture of tibia and fibula, NOS, Fracture of tibial plateau, Fracture of transverse process spine - no spinal cord lesion, Fracture of unspecified bones, Fracture of upper limb, Fracture or disruption of pelvis, Fracture tibial plateau, Fracture-dislocation or subluxation shoulder, Fractures, Fragility fracture, Fragility fracture due to unspecified osteoporosis, fragility fracture, vertebral fracture, Heel bone fracture, Hip fracture, Hip fracture NOS, Leg fracture, Malunion of fracture, Metatarsal bone fracture, Multiple fractures of foot, Multiple fractures of ribs, Multiple fractures of thoracic spine, Nonunion of fracture, Open fracture ankle, trimalleolar, Open fracture-dislocation, ankle joint, Os calcis fracture, Osteoporotic vertebral collapse, Other fracture of femur, Periprosthetic fracture, Primary open reduction fracture bone & intramedull fixation, Rib fracture NOS, Stress fracture, Temporal bone fracture, Toe fracture, Vertebroplasty of fracture of spine, Wrist fracture – closed. **Loin Pain**: Loin pain, loin pain, Left Loin Pain. **Pelvic Pain**: Pelvic pain, Pelvic and perineal pain, Pelvic mass, Acetabulum, Acetabular labrum tear, Bony pelvic pain, Other pelvic pain – female, PID, PID - pelvic inflammat disease, PID - pelvic inflammatory disease

- Neuropathy

Other chorea, Autonomic neuropathy due to diabetes, Brachial radiculitis, Meralgia paraesthetica, Mitochondrial myopathy not elsewhere classified, Morton neuroma, Morton's metatarsalgia, Myasthenia gravis, Nerve root and plexus compressions in other dorsopathies, Nerve root and plexus disorders, Nerve root or plexus disorder, Neuralgia unspecified, Neuralgia/neuritis - lower leg, Neurofibromatosis - Von Recklinghausen's disease, Neurofibromatosis type 1, Neurological symptom changes, Neuroma of amputation stump, Neuropathic pain, Numbness of hand, clonus, paraesthesia in hands, Other idiopathic peripheral neuropathy, Peripheral neuropathy, Phantom limb syndrome with pain, Polyneuropathy, Post-encephalitic syndrome, Post-herpetic neuralgia, Postherpetic neuralgia, Postzoster neuralgia, Quadriplegia, Radiculopathy, Relapsing and remitting multiple sclerosis, Right Neuropathic Pain, Secondary progressive multiple sclerosis, Sjogren - Larsson syndrome, Spasmodic torticollis, Spastic hemiplegia, Spastic paraplegia, Tetraplegia, Thoracic outlet syndrome, Transverse myelitis, Tremor symptom, Trigeminal nerve disorders, Trigeminal neuralgia, Ulnar nerve entrapment, Ulnar neuritis, Ulnar neuropathy

- Not medically related

Insurance medical, Other reasons for encounter, Global developmental delay, Other and unspecified problems related to employment, Acquired hypothyroidism, Acupuncture, Address instruction, Administration, Awaiting clinical code migration to EMIS Web, Benefits Assessed, Complaints about care, Computer summary updated, Discharge from intermediate care, Discharged from hospital, Do not attempt CPR (DNACPR) form in place, Driving licence application signed, DS1500 Disability living allowance report declined, Eligible for integrated care pathway, eMED3 (2010) new statement issued not fit for work, Failed encounter, Failed encounter - message left on answer machine, Foreign travel advice, FP10(MDA) issued, Funny turn, General builder, General chemist, Going to travel abroad, Has anticipatory care plan, History relating to military service, Homeless, Housebound, Jehovah's witness, Jury exempt form asked for, Letter encounter, MED3 issued - back to work, MED3 issued to patient, MED5 issued to patient, No follow-up, Patient self discharge, Patient's next of kin, Photosensitiveness, Poor compliance, Social problem, Social worker, Theft, Third party encounter, Vulnerable adult, Vulnerable family

- Numbness

Numbness

- Osteoporosis

Osteopenia, Acute osteomyelitis, Chronic osteomyelitis, Idiopathic osteoporosis, Idiopathic osteoporosis with pathological fracture, Osteophyte, Osteoporosis, Osteoporosis + pathological fracture lumbar vertebrae, Osteoporosis + pathological fracture thoracic vertebrae, Osteoporosis prevent, Unspecified osteomyelitis, Vertebral osteoporosis

- Pins and needles

Paraesthesia, paraesthesia, Has pins and needles, Has tingling sensation

- Prosthetic replacement

Total hip replacement, Total knee replacement, Charnley total hip replacement, Hybrid prosthetic replacement of hip joint using cement, Primary total knee replacement, Primary total prosthetic replacement of hip joint, Primary uncemented total hip replacement, Revision cemented total hip replacement, Revision of total knee replacement, Revision of total prosthetic replacement of hip joint, Revision total prosthetic replacement of shoulder joint, Thompson hemiarthroplasty of hip joint using cement, THR - Other total prosthetic replacement of hip joint, THR - Total prosthetic replacement hip joint without cement, THR - Total prosthetic replacement of hip joint using cement, TKR - Other total prosthetic replacement of knee joint, TKR - Total prosthetic replacement knee joint without cement, TKR -Total prosthetic replacement of knee joint using cement, Total hip replacement, Total knee replacement, Total prosthetic replacement of elbow joint, Total prosthetic replacement of hip joint, Total prosthetic replacement of hip joint not using cement, Total prosthetic replacement of hip joint using cement, Total prosthetic replacement of hip joint using cement, Total prosthetic replacement of knee joint using cement, Total prosthetic replacement of knee joint using cement, Total prosthetic replacement of shoulder joint, Unicompartmental knee replacement

- Referral

Neurosurgical referral, Orthopaedic referral, Patient awaiting procedure, Refer for X-ray, Refer to counsellor, Refer to geneticist, Refer to occupational therap, Refer to pain clinic, Referral for dual energy X-ray photon absorptiometry scan, Referral for echocardiography, Referral for further care, Referral to hearing aid clinic, Referral to respiratory physician, Referral to speech and language therapy service, Seen in bariatric surgery clinic, Seen in cardiology clinic, Seen in GP's surgery, Seen in hospital out-pat, Seen in neurology clinic, Seen in pain clinic, Seen in rheumatology clinic

- Repeat prescription

Issue of repeat prescription, Issue of repeat prescription for medication, Medication repeat prescript, Repeat prescription issue, Drug prescription, Medication given, Repeat medication check, Repeat prescription, Repeat prescription monitoring, Repeated prescription, Urgent request for repeat prescription

- Respiratory problems

Breathlessness, Pleuritic pain, Pulmonary nodule, Respiratory system and chest symptoms, Shortness of breath, Stridor, Acute bronchitis, Acute dry pleurisy, Acute exacerbation of asthma, Acute exacerbation of chronic obstructive airways disease, Acute infective exacerbation of chronic obstructive airways disease, Acute lower respiratory tract infection, Acute respiratory infections, Acute tracheobronchitis, Acute upper respiratory tract infection, Alpha-1-antitrypsin deficiency, Asthma, Asthma attack, Asthma, Atelectasis, Bacterial pneumonia, Bird-fancier's lung, Blood in sputum – haemoptysis, Breathless - mild exertion, Breathless - moderate exertion, Breathlessness, Bronchial asthma, Bronchiectasis, Bronchiectasis, Bronchitis unspecified, Bullous emphysema with collapse, Chronic asthma with fixed airflow obstruction, Chronic bronchitis, Chronic obst. pulm. Dis, Chronic obstr. airways disease, Chronic obstructive lung disease, Chronic obstructive pulm, Chronic obstructive pulmonary disease, Chronic obstructive pulmonary disease annual review, Chronic obstructive pulmonary disease monitoring, COAD - chr.obstr.airway dis, Compression of oesophagus, COPD self-management plan given, Difficulty breathing, Diffuse pulmonary fibrosis, Emphysema, Emphysema, Empyema, End stage chronic obstructive airways disease, Exacerbation of cystic fibrosis, Extrinsic asthma – atopy, asthma, pneumonia, pulmonary embolus, Hospital acquired pneumonia, Idiopathic pulmonary fibrosis, Interstitial lung disease, Issue of chronic obstructive pulmonary disease rescue pack, Lobar pneumonia due to unspecified organism, Lower limb spasticity, Lower resp tract infection, LTOT - Long-term oxygen therapy, Lung disease, Mild chronic obstructive pulmonary disease, Moderate chronic obstructive pulmonary disease, expiratory wheeze, Occupational asthma, Pleural effusion, Pleural plaque disease due to asbestosis, Pleurisy, Pneumonia due to unspecified organism, Pneumothorax, Pulmonary embolism, Pulmonary sarcoidosis, Recurrent bronchiectasis, Recurrent upper respiratory tract infection, Respiratory disease monitoring, Respiratory symptoms, Respiratory tract infection, Severe chronic obstructive pulmonary disease, Shortness of breath, Shortness of breath symptom, SOBOE, Suspected chronic obstructive pulmonary disease, Traumatic pneumothorax, Tuberculosis, Upper resp tract infection (GMS), Upper resp. tract infect. NOS, Upper respiratory infect.NOS, Upper respiratory infection, Upper respiratory tract infec. (GMS), Upper respiratory tract infection, Very severe chronic obstructive pulmonary disease, Viral induced wheeze, Viral upper respiratory tract infection, Wheezing, Wheezy bronchitis

- Seizure

Seizure, 2 to 4 seizures a month, Complex partial epileptic seizure, Had a fit, Hallucinations, Non-epileptic attack disorder, Partial epilepsy with impairment of consciousness

- Shingles

Shingles, Shingles, Shingles vaccination

- Sinustis

Acute frontal sinusitis, Acute maxillary sinusitis, Acute sinusitis, Acute sinusitis, Chronic sinusitis, Chronic sinusitis, Pain in sinuses, Sinus congestion, Sinusitis, Sinusitis - acute

- Skin complaints

Burning of skin, Flushing, Formication, Hyperhidrosis, Jaundice, Rash and other nonspecific skin eruption, Rash and other nonspecific skin eruption, Spots, Tingling of skin, Capillary haemangioma, Dermal naevus, Allergic skin reaction, Leg Ulcer, Leg ulcer – venous, Pressure sore, Skin of umbilicus, Bullous disorders, acne rosacea, Acne vulgaris, Acne unspecified, Actinic keratosis, Alopecia areata, Angular cheilitis, Angular stomatitis and cheilitis, Arterial leg ulcer, Asteatosis cutis, Athlete's foot, Atopic dermatitis/eczema, Balanitis, Basal cell carcinoma, Birth mark unspecified, Blepharitis, Blister of anus, Blister of foot, Blister of hand without mention of infection, Blister of lower leg, Boil, Bruise trunk, Bruises easily, Bruising symptom, Bullous pemphigoid, Burns, a rash, dry skin, hair loss, itching, Callosity on foot, Callosity under metatarsal head, Callus, Cellulitis and abscess, Cellulitis and abscess of foot, Cellulitis and abscess of leg excluding foot, Cellulitis and abscess of leg, Cellulitis and abscess of lower leg, Cellulitis and abscess of shoulder, Cellulitis and abscess of thigh, Cellulitis and abscess of toe, Cellulitis, Cellulitis of arm, Cellulitis of foot, Cellulitis of leg, Cellulitis of skin area excluding digits of hand or foot, Cellulitis, external ear, Cellulitis/abscess-forearm, Climacteric keratoderma, Cold sore (herpetic), Contact dermatitis, Contact dermatitis and other eczemas, Contact dermatitis, Corns, Corns and callosities, Cutaneous horn, Cystic acne, Darier's disease - keratosis follicularis, Dermatitis, Dermatitis/dermatoses, Dermatophytosis including tinea or ringworm, Dermatophytosis of foot, Discoid eczema, Discoid lupus erythematosus, Disseminated lupus erythematosus, Eczema, Eczemas, Erythema nodosum, Excessive sweating, Filiform wart, Fistula-in-ano, Folliculitis, Foot ulcer, Guttate psoriasis, Gynaecomastia, Haematoma of leg, Haematoma with intact skin, Hair loss, Halitosis, Hand eczema, Hand warts, Hard corn, Hidradenitis, Hidradenitis suppurativa, Hydradenitis suppurativa, Hypertrophic scar, Intertrigo, Inversion of nipple, Irritant contact dermatitis, Itch, Jaundice – symptom, Keloid scar, Laceration, Laceration – leg, Laceration, Laceration of arm, Leg bruise, Leg ulcer, Leg ulcer, Lichen planus, Lichen sclerosus et atrophicus, Melanoma in situ of back, Minor aphthous ulceration, Mole of skin, Nail clippings, Nail deformity, Nail disease, Necrotising fasciitis, Nummular dermatitis, allergic rash, bruising, cracked skin of feet, dry skin, itchy rash, rash present, skin cyst, skin lesion, skin tags, Onychogryphosis, Onycholysis, Other acne, Other specified skin disorder, Panniculitis, Papilloma of skin, Perianal irritation, Plantar fascial fibromatosis, Plantar fasciitis, Pompholyx unspecified, Porphyria cutanea tarda, Prickly heat – miliaria, Prurigo nodularis (Hyde's disease), Pruritus and related conditions, Pruritus ani, Pruritus, Pruritus vulvae, Psoriasis, Psoriasis (GMS), Psoriasis, Psoriasis-scalp-long history (GMS), Pustular psoriasis, Pyoderma gangrenosum, Recurrent boils, Rosacea, Rosacea, Scabies, Scalds, Scalp itchy, Scalp psoriasis, Scaly scalp, Sclerodactyly, Scleroderma, Seborrhoea capitis, Seborrhoeic dermatitis, Seborrhoeic dermatitis capitis, Seborrhoeic eczema, Seborrhoeic keratosis, Seborrhoeic wart, Skin care, Skin flap and skin graft operations, Skin flap, Skin lesion, Skin symptoms, Skin tag, Skin tag, Solar keratosis, Spontaneous bruising, Sunburn, Superficial pressure sore, Sweat rash, Sweating symptom, Thinning of hair, Tight foreskin, Traumatic haematoma, Tylosis palmaris et plantaris, Urticaria, Varicose eczema, Verruca plantaris, Verrucae – warts, Viral warts, Warts - viral

- Smoking

Keeps trying to stop smoking, Moderate smoker - 10-19 cigs/d, Nicotine replacement therapy, Smoking cessation advice, Smoking cessation therapy, Trying to give up smoking

- Spina Bifida

Lumbar spinal meningocele, Spina bifida, Spina bifida occulta

- Spinal stenosis

Intervertebral disc stenosis of neural canal, Spinal stenosis, Spinal stenosis, Spinal stenosis of unspecified region

- Supportive care

Supportive care

- Surgery/treatment

Amputation, Post-cardiac surgery, Postoperative care, Spinal surgery, Carpometacarpal joint of thumb, Kidney donor, Liver transplanted, Removal of orthopaedic screws, Ventriculoperitoneal shunt catheter in situ, Other fusion of spine, Abdominoplasty and liposuction, Akin's osteotomy, Ankle joint operations, Anticoagulant prophylaxis, Aortic aneurysm repair, Appendicectomy, Arthroscopic partial lateral meniscectomy, Arthroscopic partial medial meniscectomy, Arthroscopic removal of loose body from knee joint, Arthroscopic subacromial decompression, Arthroscopic total medial meniscectomy, Arthroscopic trimming of lateral meniscus, Arthroscopy, Arthroscopy, Arthroscopy of knee, Aspiration of fluid from knee joint, Bilat. salpingo-oophorectomy, Bilateral mastectomy, Bilateral vasectomy for contraception, Blind sac closure of external auditory canal, Bone graft of mandible, Bone operations, Bunionectomy, Bypass aorta anastomosis axillary artery bi femoral arteries, Bypass bifurc aorta by anastom aorta to femoral artery, Bypass of superior mesenteric artery, Carpal tunnel decompression under local (GMS), Carpal tunnel release, Cerebral artery aneurysm operations, Check cystoscopy using flexible instrument, Cholecystectomy, Cls red+int fxn proximal femoral #+screw/nail device alone, Colectomy and ileostomy, Colonoscopy abnormal, Colonoscopy planned, Colostomy, Complex reconstruction of hindfoot, Coronary art bypass graft ops, Coronary artery bypass graft operations, Correction of ptosis of eyelid, Cranioplasty using acrylic material, Creation of defunctioning ileostomy, Creation of ileostomy, Cubital tunnel release, Diagnostic arthroscopy of knee, Diagnostic arthroscopy of shoulder joint, Diagnostic colonoscopy, Diagnostic hysteroscopy and endometrial biopsy, Diagnostic laparoscopy, Diagnostic laparoscopy of female pelvis, Duodenum operations, Elective caesarean delivery, Emergency appendicectomy, Emergency caesarean section, Endarterectomy of carotid artery, Endoscopic meniscectomy of knee, Endoscopic retrograde cholangiopancreatography, Enterotomy and removal of gallstone, Epidural anaesthetic, Examination of rectum under anaesthetic, Excision biopsy of skin lesion, Excision of ganglion, Excision of ganglion of ankle, Excision of ganglion of knee, Excision of lesion of ovary, Excision of lipoma, Excision of sebaceous cyst, Excision of segment of left lower lobe, Exploratory laminectomy, Exploratory laparotomy, Exploratory thoracic laminectomy, Eye operations, Femoral hernia repair, FESS/Therapeutic endoscopy of nose and sinus, Foot joint operations, Forceps delivery, Fusion of first metatarsophalangeal joint, Fusion of first metatarsophalangeal joint of toe, Fusion of joint, Fusion of joint of cervical spine, Global parathyroidectomy, Spinal surgery, bariatric operative procedure, immunosupressive therapy, nephrectomy, splenectomy, Haemorrhoidectomy, Hallux excision arthroplasty, Hemicolectomy, Hill repair of hiatus hernia and gastropexy, Hip joint operations, Ileocaecal resection, Ileostomy formed, Implantation of cardiac pacemaker system, Implantation of dual chamber cardiac pacemaker system, Implantation of internal cardiac defibrillator, Injection of steroid into knee joint, Injection of steroid into shoulder joint, Injection of steroid into trochanteric bursa, Injection of therapeutic substance into joint, Insertion of vagal nerve stimulator, Internal fixation of bone, Intramuscular injection, Introduction of tension free vaginal tape, Jaw and temporomandibular joint operations, Knee joint operations, Knee: meniscectomy, Lambrinudi Operation Right, Laminectomy, Laminectomy approach to lumbar spine, Laminectomy approach to thoracic spine, Laminectomy. (GMS), Laparoscopic bilateral female sterilisation, Laparoscopic cholecystectomy, Laparoscopic gastric bypass, Laparoscopic Nissen fundoplication using abdominal approach, Laparotomy, Large loop excision transformation zone, Left hemicolectomy, Left hemiparesis, Left salpingoophorectomy, Lobectomy of lung, Localised fusion of joints of hindfoot, Localised fusion of joints of midfoot and forefoot, Lower uterine segment caesarean section (LSCS), Lumbar facet joint injection, Lumpectomy of breast, Mastectomy of left breast, Mastectomy of right breast, Minor surg done – cryotherapy, Minor surgery done, Minor surgery done – cautery, Minor surgery done – injection, Minor surgery done – other, Minor surgery done + claimable, Mitral valve repair, Mitral valve replacement (GMS), Monk hemiarthroplasty hip, Nasal polypectomy, Nasal polyps, Nasojejunal feeding, Nephrectomy, Nephrostomy, Nerve block NEC, Non obstetric encircling suture of cervical os, Nose operations, Nursing care – injections, Operation on intervertebral disc, Operations on hydrocele, Operations, procedures, sites, OS other primary decompression operations on lumbar spine, Osteotomy, Osteotomy of bone of foot, Osteotomy of first metatarsal, Osteotomy of foot, Other arthroplasty, Other bypass of femoral artery or popliteal artery, Other caesarean delivery, Other fixation of bone, Other graft of bone, Other laparoscopic female sterilisation, Other open pyeloplasty, Other operations on bowel, Other operations on haemorrhoid, Other primary fusion of joint, Other prosthetic hemiarthroplasty of hip, Other reconstruction of ligament, Other right hemicolectomy, Other specified cemented hemiarthroplasty of shoulder, Other specified operations on foot joint, Other specified operations on shoulder joint, Other specified primary lumbar discectomy, Other specified repair of recurrent incisional hernia, Other total prosthetic replacement of hip joint, Other total prosthetic replacement of joint, Other total prosthetic replacement of knee joint, Ovarian cystectomy, Panproctocolectomy, Parastomal hernia, Partial gastrectomy, Partial lobectomy of lung, Pelvic floor repair, Percut transluminal balloon angioplasty one coronary artery, Percutaneous transluminal angioplasty of femoral artery, Phacoemulsification lens insertion prosthetic replacement, Pilonidal sinus operations, Plastic repair of quadriceps tendon, Plastic repair of rotator cuff of shoulder, Plastic surgery, Pneumococcal vaccination given, Pneumonectomy operations, Post operative monitoring, Post-operative pain, Posterior repair, Postoperative complication, Postoperative pain, Postoperative seroma, Postsurgical hypothyroidism, Prim anterior cervical spine corpectomy reconstruction HFQ, Prim post interspin lumb fuse, Primary anterior excis cervical IV disc & interbody fusion, Primary arthrodesis of joint NEC, Primary cemented hemiarthroplasty of hip, Primary cemented total knee replacement, Primary decompress thoracic spinal cord fusion thorac spine, Primary decompression operation on cervical spine, Primary decompression operations on lumbar spine, Primary fusion of joint of lumbar spine, Primary inguinal hernia repair, Primary laminectomy excision of cervical intervert disc, Primary laminectomy excision of lumbar intervertebral disc, Primary laparoscopic repair of inguinal hernia, Primary lumbar discectomy, Primary lumbar microdiscectomy, Primary microdiscectomy of lumbar intervertebral disc, Primary posterior fusion of lumbar spine, Primary repair of tendon, Primary transforaminal interbody fusion joint lumbar spine, Prmy open red+int fxn prox femoral #+screw/nail+plate device, Prmy open reduction of #+internal fixation with plate, Prosthetic replacement of mitral valve, Prosthetic uncemented hemiarthroplasty of shoulder, Proximal row carpectomy, Radical hysterectomy with conservation of ovaries, Radical nephrectomy, Radical prostatectomy without pelvic node excision, Reconstruction of anterior cruciate ligament of knee, Release of trigger finger, Removal of gastric band, Removal of plate from bone, Repair of recurrent incisional hernia, Repair of umbilical hernia, Replacement of aortic valve, Replacement of aortic valve, Resurfacing arthroplasty, Resurfacing of joint, Reversal of Hartmann's procedure, Reversal of ileostomy, Revision cemented hemiarthroplasty of shoulder, Revision of bypass for coronary artery, Revision of fundoplication of stomach, Revision uncemented hemiarthroplasty of hip, Revisional lumbar discectomy, Revisional lumbar microdiscectomy, Rhinoplasty, Right salpingoophorectomy, Rigid oesophagoscopic dilation of oesophagus, Rotator cuff decompression - open acromioplasty, Sampling of axillary lymph nodes, Septoplasty of nose, Septorhinoplasty, Shoulder joint operations, Shoulder joint operations, Sigmoid colectomy, Simple arthrodesis, Simple mastectomy, Simple nephrectomy – other, Splenectomy, Standard circumcision, Subacromial decompression, TAH - Tot abdom hysterectomy and BSO - bilat salpingophorect, Tenotomy, Therapeutic arthroscopic operations on cavity of knee joint, Thoracoscopic video-assisted approach to thoracic cavity, Tibial osteotomy, Total cholecystectomy, Total colectomy, Total gastrectomy, Total lobectomy of left lower lobe, Total lobectomy of right upper lobe, Total nephrectomy, Total nephrectomy, Total splenectomy, Tracheostomy, Transplantation of liver, Transurethral prostatectomy, Trapeziumectomy, Traumatic arthropathy of shoulder, Triple therapy helicobacter pylori, TURBT - Transurethral resection of bladder tumour, Tympanoplasty, Uci total replacement of knee joint using cement, Unilateral recurrent inguinal hernia – simple, Ureteroscopy, Vasectomy requested, Ventriculocisternostomy, Whipple pancreaticoduodenect, Whipple pancreaticoduodenectomy

- Suspected condition

Suspected condition

- Swelling/inflammation

Dependent oedema, Peripheral oedema, Acquired (chronic) lymphoedema, Acute prostatitis, Baker's cyst, Bloating symptom, scrotal swelling, a swelling, Finger swelling, Ganglion of foot, Ganglion of wrist, Ganglion unspecified, Leg swelling, Leg swelling symptom, ankle oedema, leg oedema, oedema not present, oedema of ankles, oedema of feet, oedema of legs, scrotal swelling, submandibular swelling, Oedema, Osgood schlatter's dis, Osgood-Schlatter's dis - osteochondrosis of tibial tubercle, Pitting oedema, Popliteal bursitis, Reactive arthropathy unspecified, Salivary gland disease, Swelling, Swelling of calf, Swollen calf, Swollen foot, Swollen hand, Swollen joint, Swollen knee, Swollen legs, Swollen lower leg, Swollen nose, Swollen thumb, Ulcerative colitis, Ulcerative colitis and/or proctitis, Wegener's granulomatosis

- Testing

Abnorm.liver function test, Abnormal liver function test, LFT's abnormal, Adult screening, Angiogram, Angular cheilitis, Awaiting results, Blood sample taken, Blood test due, Breast examination, Computerised tomograph scan, CT scan brain – normal, ECG abnormal, ECG: Q-T interval prolonged, Echocardiogram, Echocardiogram abnormal, Echocardiogram normal, Helicobacter breath test, HEp-2 cell autoantibody screening test, Inform patient of results, Investigation result, Laboratory test requested, Liver function test, Liver function tests, Liver function tests abnormal, Magnetic resonance imaging of lumbar spine, Magnetic resonance imaging of lumbar spine abnormal, MRI of knee, MRI of lumbar spine, MRI of shoulder, MRI scan abnormal, Partially informed of test results, Patient informed - test result, Plain X-ray abdomen, Plain X-ray hand, Plain X-ray knee normal, Platelet count abnormal, Scaphoid X-ray, Screening, Standard chest X-ray, Standard chest X-ray abnormal, Test result to pat.by 'phone, Test result to pat.personally, Test result to patient, Thyroid function test, Thyroid function tests abnormal, Tuberculosis screening, U-S gallbladder scan, U-S pelvic scan

- Therapeutic prescription

Therapeutic prescription

- Thyroid issues

Acquired hypothyroidism, Thyroid disorder, Graves' disease, Hyperthyroidism, Hypopituitarism, Hypothyroidism, Hypothyroidism, Nontoxic multinodular goitre, Subclinical hypothyroidism, Thyrotoxicosis, TSH level

- Tiredness or sleep

Drowsiness, Fatigue, Lassitude, Lethargy, Post polio exhaustion, Sleep disturbances, Sleeping problem, tired all the time, Cannot sleep – insomnia, Chronic fatigue syndrome, Chronic fatigue syndrome, Excessive sleep, Fatigue, Insomnia, Insomnia symptom (GMS), Lethargic, Lethargy – symptom, Myalgic encephalomyelitis, Obstructive sleep apnoea, Persistent insomnia, Poor sleep pattern, Sleep apnoea, Sleep disorders, Snoring symptoms, Tired all the time, Tiredness symptom, Transient insomnia

- Tumour

Adenoma, Aggressive fibromatosis, Carcinoid tumour, Carcinoid tumours, Cartilaginous exostosis, Melanocytoma of eyeball, Meningiomas, Neuroendocrine carcinoma, Neuroendocrine neoplasm, Neurofibroma, Neurofibromas, Oligodendroglioma, Osteoma, Paraganglioma, Schwannoma, Acoustic neuroma, Benign neoplasm of spine, Cerebral meningioma, Lipoma, Lipoma of abdominal wall, Lipoma of back, Liver metastases, Multiple congenital exostosis, Parotid lump, Phaeochromocytoma, Pituitary adenoma, Sacrococcygeal disorders not elsewhere classified, Spinal meningioma

- Urinary system complaints

Urgency of micturition, Acute retention of urine, Albuminuria, Fowler's Syndrome, Incontinence of urine, Microalbuminuria, Raised PSA, Renal colic, Retention of urine, Slowing of urinary stream, Continence assessment, Urinary incontinence, Other specified disorders of bladder, Urolithiasis, Acquired cyst of kidney, Acute kidney injury, Acute kidney injury stage 1, Acute kidney injury stage 3, Acute pyelonephritis, Attention to urinary catheter, Benign prostatic hypertrophy, Bladder calculus, Bladder disorders, Bladder outflow obstruction, Blood in urine – haematuria, Burch colposuspension, Calculus of kidney, Catheter complications, Cauda equina syndrome, Chronic cystitis, Chronic interstitial cystitis, Chronic kidney disease, Chronic kidney disease stage 2, Chronic kidney disease stage 3, Chronic kidney disease stage 3A without proteinuria, Chronic kidney disease stage 3B, Chronic kidney disease stage 3B with proteinuria, Chronic kidney disease stage 4, Chronic kidney disease stage 5, Chronic renal failure, CKD stage 3 with proteinuria, CKD stage 3A with proteinuria, CKD stage 3B without proteinuria, CKD stge 3A without proteinuria, CKD with GFR category G2 & albuminuria category A1, CKD with GFR category G3a & albuminuria category A1, Cystitis, Degree of urinary incontinence, Detrusor instability, Dysuria, End stage renal failure, Frank haematuria, Frequency of micturition, renal disease, Haematuria, Hydronephrosis with pelviureteric junction obstruction, Impaired renal function disorder, Incontinence of urine, Indwelling urethral catheter, Irritable bladder, Kidney calculus, Lower urinary tract symptoms, Microscopic haematuria, Micturition frequency, Micturition stream, Mild lower urinary tract symptoms, Nocturia, Nocturnal enuresis, Nonspecific urethritis, kidney stone, Overactive bladder, Polycystic kidney disease, Polycystic kidney disease, Polyuria, Prostatism, Prostatitis, Puerperal endometritis, Pyelonephritis unspecified, Recurrent urinary tract infection, Recurrent UTI, Reflux – vesicoureteric, Renal calculus, Renal calculus, Renal colic, Renal dialysis, Renal function monitoring, Renal haematoma without mention of open wound into cavity, Renal impairment, Renal profile, Renal stone, Stress incontinence, Stress incontinence – female, Suspected UTI, Transitional cell papilloma of bladder, Unstable bladder, Ureteric colic, Ureteric stone, Urethral diverticulum, Urge incontinence of urine, Urgency of micturition, Urinary frequency, Urinary symptoms, Urinary tract infection, Urinary tract infection, site not specified, Urinary tract infection site not specified,

- Vertigo

Acute vertigo, Dizziness, Light-headedness, Vertigo NOS, Benign paroxysmal positional vertigo, Benign paroxysmal positional vertigo or nystagmus, Dizziness symptom, Feels off balance

- Viral illness

Viral illness

- Weight issues

Abnormal loss of weight, Abnormal weight gain, Abnormal weight loss, Abnormal weight loss – symptom, Body mass index 30+ - obesity, Body mass index 40+ - severely obese, Complaining of weight loss, Health education - weight management, Morbid obesity, Under weight, Obese class III (BMI equal to or greater than 40.0), Obesity, Obesity hypoventilation syndrome, Obesity monitoring, Wants to lose weight, Weight decreasing, Weight increasing, Weight monitoring, Weight symptom

- Whiplash injury

Whiplash injury

- Wound care

Pressure sore, Wound management, Open wound of other parts of hip and thigh, Dressing of wound, wound healing, wound necrotic, Open wound of finger(s), Open wound of leg, Open wound of lip, Post-operative wound care, Pressure sore, Venous ulcer of leg, Wound care, Wound observation

- Other

Dizzyness, History/symptoms, Breast Disorders, Syncope, Unsteady symptom, Spleen enlargement Clinical Opiate Withdrawal Scale, Down's Syndrome, Lack of Libido, Medically unexplained symptoms, Sicca (Sjogern's) syndrome, Anosmia loss of smell, Blackout, Electric Shock, Addison's Disease, Analgesia Present, Angiotensin converting enzyme inhibitor not tolerated, Ascites, Autism, Best interest decision made on behalf of patient, Cold Hands, Gender reassignment patient, General symptoms, Loss of appetite, Raised immunological level, Serious Diagnosis, Statin decline
