# Supplementary material for: Opioid prescribing and social deprivation: A retrospective analysis of prescribing for CNCP in Liverpool CCG
Source: PLoS One. 2023 Mar 8;18(3):e0280958. doi: 10.1371/journal.pone.0280958 (PMC9994720; doi:10.1371/journal.pone.0280958)
Supplement: S3 File — (DOCX) [file pone.0280958.s003.docx]

# Table S3a: Average (median) length of opioid prescriptions contributing to daily doses exceeding 120mg MED.

| **Opioid** | **No. of Prescriptions** | **Median Duration (weeks)** | **Range** |
| --- | --- | --- | --- |
| Morphine | 760 | 79.71 | 1017.43 |
| Tramadol | 568 | 88.71 | 905 |
| Oxycodone | 525 | 97.29 | 677.14 |
| Codeine | 461 | 106.14 | 1078 |
| Fentanyl | 290 | 91.21 | 568.43 |
| Buprenorphine | 282 | 82.5 | 900 |
| Dihydrocodeine | 83 | 104.43 | 1063.14 |
| Methadone | 19 | 2.86 | 256.57 |
| Tapentadol | 7 | 79.9 | 117.71 |
| Pethidine | 3 | 63.71 | 221.29 |
| Hydromorphone | 1 | 252.43 | 0 |

# Table S3b: Average (median) length of opioid prescriptions contributing to **average** daily doses exceeding 120mg MED.

| **Opioid** | **No. of Prescriptions** | **Median Duration (weeks)** | **Range** |
| --- | --- | --- | --- |
| Morphine | 69 | 175 | 621.71 |
| Tramadol | 20 | 183.93 | 673.71 |
| Oxycodone | 155 | 110.14 | 677.14 |
| Codeine | 43 | 133 | 824.57 |
| Fentanyl | 209 | 91.00 | 567.43 |
| Buprenorphine | 96 | 76.42 | 900 |
| Dihydrocodeine | 7 | 147.71 | 832.39 |
| Hydromorphone | 1 | 252.43 | 0 |
| Pethidine | 1 | 285 | 0 |
